# Therapeutic DNA Vaccination of Vertically HIV-Infected Children: Report of the First Pediatric Randomised Trial (PEDVAC)

**DOI:** 10.1371/journal.pone.0079957

**Published:** 2013-11-28

**Authors:** Paolo Palma, Maria Luisa Romiti, Carla Montesano, Veronica Santilli, Nadia Mora, Angela Aquilani, Stefania Dispinseri, Hyppolite K. Tchidjou, Marco Montano, Lars E. Eriksson, Stefania Baldassari, Stefania Bernardi, Gabriella Scarlatti, Britta Wahren, Paolo Rossi

**Affiliations:** 1 University Department of Pediatrics, Unit of Immune and Infectious Diseases, Children's Hospital “Bambino Gesu”, Rome, Italy; 2 Department of Medicine, Chair of Pediatrics, University of Rome “Tor Vergata”, Rome, Italy; 3 Department of Biology, University of Rome “Tor Vergata”, Rome, Italy; 4 Unit of Viral Evolution and Transmission, San Raffaele Scientific Institute, Milan, Italy; 5 Chair of Infectious Diseases, University of Rome “Tor Vergata”, Rome, Italy; 6 Department of Neurobiology, Care Sciences and Society, Karolinska Institutet and Department of Infectious Diseases, Karolinska University Hospital, Huddinge, Sweden; 7 School of Health Science, City University, London, United Kingdom; 8 Department of Microbiology, Tumor and Cell Biology, Karolinska Institutet, Stockholm, Sweden; University of New South Wales, Australia

## Abstract

**Subjects:**

Twenty vertically HIV-infected children, 6–16 years of age, with stable viral load control and CD4+ values above 400 cells/mm^3^.

**Intervention:**

Ten subjects continued their ongoing antiretroviral treatment (ART, Group A) and 10 were immunized with a HIV-DNA vaccine in addition to their previous therapy (ART and vaccine, Group B). The genetic vaccine represented HIV-1 subtypes A, B and C, encoded Env, Rev, Gag and RT and had no additional adjuvant. Immunizations took place at weeks 0, 4 and 12, with a boosting dose at week 36. Monitoring was performed until week 60 and extended to week 96.

**Results:**

Safety data showed good tolerance of the vaccine. Adherence to ART remained high and persistent during the study and did not differ significantly between controls and vaccinees. Neither group experienced either virological failure or a decline of CD4+ counts from baseline. Higher HIV-specific cellular immune responses were noted transiently to Gag but not to other components of the vaccine. Lymphoproliferative responses to a virion antigen HIV-1 MN were higher in the vaccinees than in the controls (p = 0.047), whereas differences in reactivity to clade-specific Gag p24, RT or Env did not reach significance. Compared to baseline, the percentage of HIV-specific CD8+ lymphocytes releasing perforin in the Group B was higher after the vaccination schedule had been completed (p = 0.031). No increased CD8+ perforin levels were observed in control Group A.

**Conclusions:**

The present study demonstrates the feasibility, safety and moderate immunogenicity of genetic vaccination in vertically HIV-infected children, paving the way for amplified immunotherapeutic approaches in the pediatric population.

**Trial registration:**

**clinicaltrialsregister.eu** _2007-002359-18 IT

## Introduction

Current antiretroviral therapy (ART) recommended for the HIV-infected pediatric population requires medication early, daily and indefinitely [1]. The life-long need for strict adherence and the costs of chronic treatment encourage the exploration of alternative approaches that could potentially be complementary to long-term management of pediatric HIV infection. Young children who are highly adherent to ART frequently encounter adherence problems during adolescence [1]. Recent data clearly show that vertically HIV-infected children have a progressively increased risk of developing triple-class virological failure after 5 years on highly active ART [2]. Thus, new therapeutic strategies are necessary for the pediatric population, particularly as these children approach adolescence. Transient decay of latently HIV-infected CD4+ T-cells has been reported after a therapeutic vaccination with a HIV-recombinant poxvirus [3]. Furthermore, T-cell induced HIV viral modifications have been reported with an adenovirus-based prophylactic HIV vaccine in the STEP trial [4]. These data demonstrate the possibility of exerting selective viral pressure with a vaccine strategy. However, no data on immunotherapeutic HIV vaccine strategies are yet available for children. Here we report data from the PEDVAC trial, which is the first study of an HIV-DNA therapeutic vaccine in vertically infected children.

## Materials and Methods

### Study subjects and trial design

**Figure 1:**
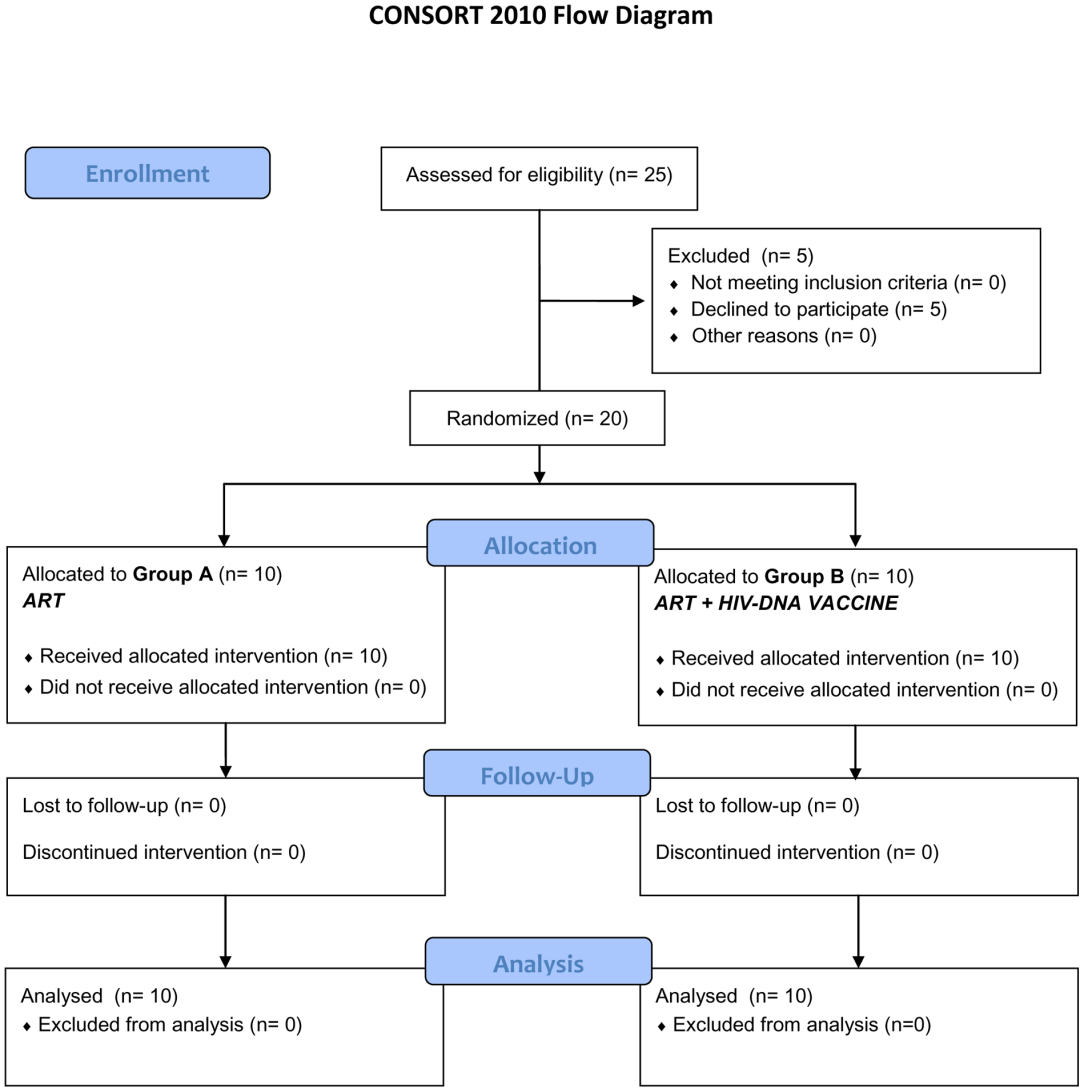
CONSORT 2010 flow diagram.

The PEDVAC trial is a single center, phase II open label randomized trial, laboratory blinded, which evaluates feasibility, safety and immunogenicity of a multiclade, multigene HIV-DNA vaccine (HIVIS) [5]. The trial was judged as a phase IIa trial by the Italian regulatory Agencies (AIFA) according to the definition by the National Institutes of Health, Bethesda, USA: “*pilot, proof of concept clinical trials to evaluate efficacy (and safety) in selected populations of patients*” [6]. HIV vertically infected children (4–16 years of age), on stable antiretroviral regimen for at least 6 months with HIV-RNA<50 copies/ml and stable CD4+ counts (≥400 cells/mm^3^ or 25%) over 12 months of follow-up, were eligible for the study. Patients with ongoing other infections or on treatment with immunomodulatory agents or with signs or history of autoimmune diseases were excluded from the study. Between January and September 2009, 25 vertically HIV-infected children were screened for this study, 20 of whom were enrolled: 10 in each of Groups A and B (Figure 1). Enrolled patients were randomized at week −2 into two groups: a control group of 10 children who continued previous antiretroviral regimen (control Group A) and a group of 10 children immunized intramuscularly with the HIV-DNA vaccine, in addition to their previous and ongoing antiretroviral regimen (vaccine Group B). Clinical and laboratory evaluations were performed at baseline (weeks −2 and 0) and at weeks 4, 12, 16, 20, 36, 40, 48, 60, 72, 84 and 96. In addition, HIV viral load, lymphocyte subsets, anti-ANA and anti-dsDNA antibodies were performed at each time point [5]. Immunizations by intramuscular injection were scheduled at weeks 0, 4 and 12, with a boosting dose at week 36. All vaccine doses were administered at the Clinical Trial Center Unit, “Bambino Gesù” Children's Hospital, Rome, Italy. ART was continued in all patients during the immunization schedule and for the duration of the study. The protocol for this trial and supporting CONSORT checklist are available as supporting information; see Checklist S1 and Protocol S1.

### Ethical approval

The trial Eudract number 2007-002359-18 was approved by the Ethical Committee of the Children's Hospital “Bambino Gesù” and by the Italian Regulatory Agency for Drug Administration (AIFA), and conducted in accordance with the Declaration of Helsinki. Written informed consent was approved by the Ethical Committee of the hospital and obtained from all participants and from their guardians. All the source documents, including each patient's case report form (CRF), have been stored in a locked secure area at the Clinical Trial Center of the hospital.

### Randomization and blinding

The randomization schedule was prepared by the statisticians at the Clinical Trial Center of “Bambino Gesù” in Rome. The randomization list was performed by Electronic Software available at RANDOM.ORG. Laboratory staff remained blinded with respect to the allocation of control or vaccine group.

### Vaccine and formulation

Two preparations of HIV-DNA plasmids have been prepared for the HIVIS vaccine: (1) DNA plasmids (2 mg/ml) encoding HIV Env A, B, C and Rev B [Ampoule 1, 0.7 ml *pKCMV* (*rev, envA, envB, envC*)]; (2) DNA plasmids (2 mg/ml) encoding HIV Gag A, B and RT mut B [Ampoule 2, 0.5 ml *pKCMV* (*gagA, gagB, RTmut*)]. *In vitro* each plasmid expresses 10–100 fg protein/transfected cell when delivered separately, *in vivo* in mice electroporation or Bioject delivery enhances immunogenicity around 100-fold [8], [9], [10].The vaccine was manufactured in accordance with Good Manufacturing Practice by Vecura (Karolinska Institutet and University Hospital at Huddinge, Stockholm, Sweden). It was approved by the Medical Products Agency (Läkemedelsverket) for a prophylactic HIV vaccine study of 40 healthy adults in Sweden [7], [8] and by the Italian Regulatory Agency for Drug Administration (AIFA) for the present study. The vaccine was not adjuvanted following results obtained in healthy adult volunteers, where higher cellular responses were detected without the administration of granulocyte macrophage colony stimulating factor (GM-CSF) as adjuvant [7]. Each child received 4 ampoules at each vaccination visit (2 of ampoule no. 1 in the right arm and 2 of ampoule no. 2 in the left arm), amounting to a total of 3.8 mg DNA, by intramuscular injection in the two deltoid sites. Division of plasmids into two lots was done to avoid immunodominance of either composition [9].

### Safety evaluation

Physical and laboratory safety evaluations were performed as previously described [5]. All medical events were recorded as possible adverse events and were graded by the principal investigator as to their severity and relationship to the immunization. Immediate local (erythema, induration, pain, regional lymphadenopathy, restricted movement) and systemic (headache, nausea, fatigue, fever, gastrointestinal upset, vomiting, diarrhoea, myalgia, hypersensitivity) reactions were directly observed during the first hour after the HIV-DNA application. HIV-infected children or their parents recorded in a self-administered diary card the occurrence and severity of solicited local and systemic events, using a standard scale to grade reactions [5].

### Lymphoproliferation assay (LPA)

Fresh peripheral blood mononuclear cells (PBMC) were analyzed by lymphoproliferation assay as previously described [5], [11]. Briefly, 2×10^5^ PBMC were cultured in 96 well plates for 7 days with the following antigens: aldrithiol-2 (AT-2)-treated HIV-1 MN virions subtype B at 2.5 µg/ml and non-viral control antigen SUPT1 microvesicles at 2.5 µg/ml (kindly provided by Dr. J. Lifson, SAIC Frederick, Inc., Frederick, USA) recombinant proteins rgp160 at 0.5 µg/ml (Protein Sciences, Meriden, CT, USA), Reverse Transcriptase (rRT) subtype B at 0.5 µg/ml, rgp41 at 0.5 µg/ml, rp24 0.5 at µg/ml, and recall antigens Cytomegalovirus (CMV) at 0.5 µg/ml and Candida Albicans at 0.5 µg/ml (Nanogen TO, IT). Results are shown as mean values of duplicates and are expressed as counts per minute (cpm). Stimulation index (SI) was defined as previously described [5], [11]. SI was considered as positive when ≥3. For stimulation by HIV-1 MN inactivated virions, the cut off was set at SI 15, since lymphocytes from non-infected controls at times reacted with both the SUP T1 control and HIV-1 MN preparations.

### Intracellular cytokine staining assay

Heparinized fresh whole blood (100 µl) diluted 1∶2 in complete RPMI medium was incubated with 1 µg each of anti-CD28 and anti-CD49d monoclonal antibodies (mabs) and 1 µg of a protein pool consisting of recombinantly produced proteins representing the vaccine sequences HIV-1 RT (Clade B), HIV-1 p17/24 (Clade B), HIV-1 p17/24 (Clade C), HIV-1 UG37 gp140 (Clade A) and CN54 gp140 (Clade C) or controls (anti-CD49d and anti-CD28 mabs) for 16 h. After 2 h, brefeldin A (Sigma-Aldrich, St. Louis, MO) was added, 10 µg/ml. After 14 h, cells transferred to 5 ml tubes were stained with anti-CD3, anti-CD4 and anti-CD8 antibodies. Red blood cells were lysed with BD FACS Lysing solution (BD, San Josè, CA), fixed with 4% paraformaldehyde and washed with PBS with 1% bovine serum albumin (BSA). Lymphocytes were stained with anti IFN-γ mab in PBS with 1% BSA and 0.5% saponin. Cells resuspended in Facs Flow were analyzed with a FacsCanto II. Data files were analyzed using DIVA software (BD) and results expressed as percentage of cytokine-producing CD3+, CD4+ and CD8+ T-cells. Results were considered positive when the percentage of cytokine-secreting cells of HIV-stimulated cultures was higher or equal to 0.02% above unstimulated cultures and were considered assessable if the percentage of cytokine-producing control cells was lower than 0.06% [5], [12].

### TZMbl neutralization assays and preparation of pseudoviruses

Pseudovirus (PSV) of HIV-1 strain SF162 was prepared by transfection of 293T cells as previously described [13]. To circumvent the effect of reverse transcriptase (RT) inhibitors present in sera, we utilized an RT-resistant backbone vector, 1617RT/K103N, which carries mutations in the pol gene conferring resistance to nucleoside and non-nucleoside reverse transcriptase inhibitors. SF162 PSV-1617RT/K103N tested with antiretroviral drugs Efavirenz, NVP, Zidovudine, Saquinavir and Raltegravir, showed that Efavirenz and Raltegravir were the only drugs still interfering in the TZMbl assay. To further exclude unspecific inhibiting effects, sera were also tested against the vesicular stomatitis (VSV) pseudovirus. The PSVs and TZMbl neutralization assays were performed with a previously described protocol using four steps of 4-fold dilutions starting with 1∶20 of each serum [14].

### Plasma HIV-RNA determination

Plasma HIV-RNA was determined using a quantitative b-DNA assay (Quantiplex HIVRNA 2.0 bDNA Assay, Chiron Diagnostics Corporation, Emerville, CA, USA) with a lower limit quantification of 50 copies/ml of plasma.

### Quantification of residual plasma HIV-RNA viremia

Plasma obtained from blood sampled in EDTA was stored frozen at −80°C until tested. Residual viremia was quantified by an ultrasensitive assay, based on Amplicor HIV-1 Monitor v1.5 (Roche Molecular Systems, USA) with a limit of detection of 1 copy/ml. Modifications include pelleting of the virus from 2 ml or more of plasma at 200.000 g at 4°C for 1 h. The HIV-RNA pellet is extracted by adding half of the normal volume of quantification standard, suspended in 1/8 of standard volume diluents, and assayed by reverse transcription and polymerase chain reaction (PCR). PCR and detection steps follow the manufacturer's protocol. To calculate HIV-RNA copy number, we adjusted the correction factor according to the modified volume of the internal quantification standard.

### Cell-Associated HIV-DNA quantification

Total cell DNA is extracted from PBMC pellets with High Pure PCR Template Preparation Kit (Roche Molecular Biochemicals, Germany) and stored at −20°C. Total HIV-DNA is quantified by real-time polymerase chain reaction, using 5′ nuclease assay in the long terminal repeat (LTR) region of proviral HIV-1 (reference sequence HXB2) performed on a LightCycler v3.5 (Roche Molecular Biochemicals, Indianapolis, IN). Up to 500 ng of DNA is amplified with sense primer NEC 152 and reverse primer NEC 131, producing a 121 bp fragment in the presence of a dually (FAM and TAMRA) labelled NEC LTR probe [15], [16] and read on channel F1/F2 of the Light Cycler. The PCR sensitivity was 1 copy of HIV-DNA/reaction (approximately equal to 10 copies/10^6^ PBMC's of total HIV proviral DNA). To verify DNA integrity we used LC control DNA Kit reagents (Roche Molecular Biochemicals, Germany) to amplify a 110 bp fragment of human β-globin in the same reaction. This internal control target hybridises with a FRET probe and is read on channel F3/F2. To verify the accuracy of the Real-Time PCR result, different HIV-1 DNA standards were also quantified [17]. Results are expressed as number of total HIV-proviral DNA copies/10^6^ PBMC.

### Psychological impact of the PEDVAC trial

Difficulties in being part of a therapeutic vaccine trial and its impact on adherence to treatment were assessed from week 0 onwards by a specifically developed questionnaire adapted from WHOQOL-HIV and PENTA (Pediatric European Network for Treatment of AIDS), as previously described [12]. Before study entry, the aim of the study was extensively explained to the eligible patients and their parents by the personal physicians and a psychologist. Questionnaires were filled in at the time of scheduled visits to the clinic by parents or caregivers and patients with the help of a medical psychologist.

### Statistical analysis

The present study was intended as an exploratory proof of concept to test feasibility and immunogenicity of therapeutic DNA vaccination in vertically HIV infected children. With currently available antiretroviral regimes, there are good possibilities to prevent mother-to-child viral transmission when the HIV infection of the mother is known. Consequently there is a low incidence of vertical HIV infection in children where treatment of mother and newborn is available. Therefore, we had a limited number of children with vertical HIV infection available. For practical reasons and in concert with the ethical committee of the Children's Hospital “Bambino Gesù” we chose to recruit 20 children to randomize to receive DNA vaccine or to serve as controls, both groups receiving standard antiretroviral treatment. Study data were entered under study code and initials on clinical report forms. Clinical and vaccine safety laboratory data were entered in an Access (Microsoft) database. Statistical calculations were performed in IBM SPSS Statistics, version 19 (SPSS, Inc.) and GraphPad Prism Software version 5.00. Statistical significance was accepted if p≤0.05. As regards calculations on the immunological outcome measures, the area under the curve (AUC) methodology of responses for the whole follow up period were chosen as a summary measure as suggested by Matthews et al (18). AUC was chosen because the biological responses to HIV were of peaked manner and there were unequal time intervals between testing. As regards cellular immune reactivity from lymphocyte proliferations, intracellular assays and HIV-RNA viral load values (including ultrasensitive HIV-RNA), AUC [18] were calculated from baseline to week 96 for each patient and potential differences between the vaccinated and control groups were analysed by independent samples t-test. With this selected approach, we had power to detect only larger magnitudes of between group differences as regards the AUC summary measures of immune responses. With 10 subjects in each group, an effect size of 1.32 was needed for a power of 0.8 to detect a statistically significant difference between the groups by t-test, this transfers to a critical t of 2.1 with the variance of the existing data, this means for example a mean difference of over 278 SI for the mean MN data and a mean difference of over 46 SI for the RT data. The within group difference in the intracellular staining assays at weeks 0 vs 60 was analyzed by Wilcoxon signed-rank test for the vaccinated and control groups respectively. Differences in rate of adherence during the study were calculated by significance test for a difference in two proportions [19]. Comparison between the vaccinated and control groups for HIV-DNA values was calculated by Mann-Whitney U test. The relationship of HIV antigens p24 and HIV-MN reactivity was calculated by Spearman's rank correlation on samples at week 16.

## Results

### Patients enrolled

Five patients withdrew after viewing the follow-up flowchart (Figure 1). Failures during screening were due to the lack of consent to planned additional visits. Baseline characteristics of 20 enrolled children are reported in Table 1. All patients were long-term virally suppressed before study entry, vaccinees during a median of 69 months (range 12–137) and the controls for a median of 101 months (range 13–156). Median time and range of treatment with the same ART regimen is also reported in Table 1. Four enrolled patients were treated early with ART (within the first year of life) and were equally randomized to the two groups. All 10 patients in Group B completed the vaccination schedule.

**Table 1:** Patient data at enrolment.

	GROUP A Controls	GROUP B Vaccinees
**Female/Male**	6/4	6/4
**Age (years) median (range)**	12,0 (8,1–16,3)	11,5 (6,3–14,3)
**CD4+ percentage median (range)**	35,5 (28–47)	34 (28–42)
**CD4+ no. of cells/mm^3^, median (range)**	748,5 (423–1188)	798 (497–1094)
**Median time in months with HIV<50 copies/ml before study entry (range)**	101 (13–156)	69 (12–137)
**ART: 2 NRTI/PI**	5/10	4/10
**ART: 2 NRTI/NNRTI**	5/10	6/10
**Median time in months with the same ART (range)**	12 (12–42)	16,5 (9–46)
**Early ART treated children within the first year of life**	2/10	2/10

ART signifies antiretroviral treatment with PI = protease inhibitors, NRTI = nucleoside reverse transcriptase inhibitors, NNRTI = non nucleoside reverse transcriptase inhibitors.

### Vaccine safety

**Figure 2:**
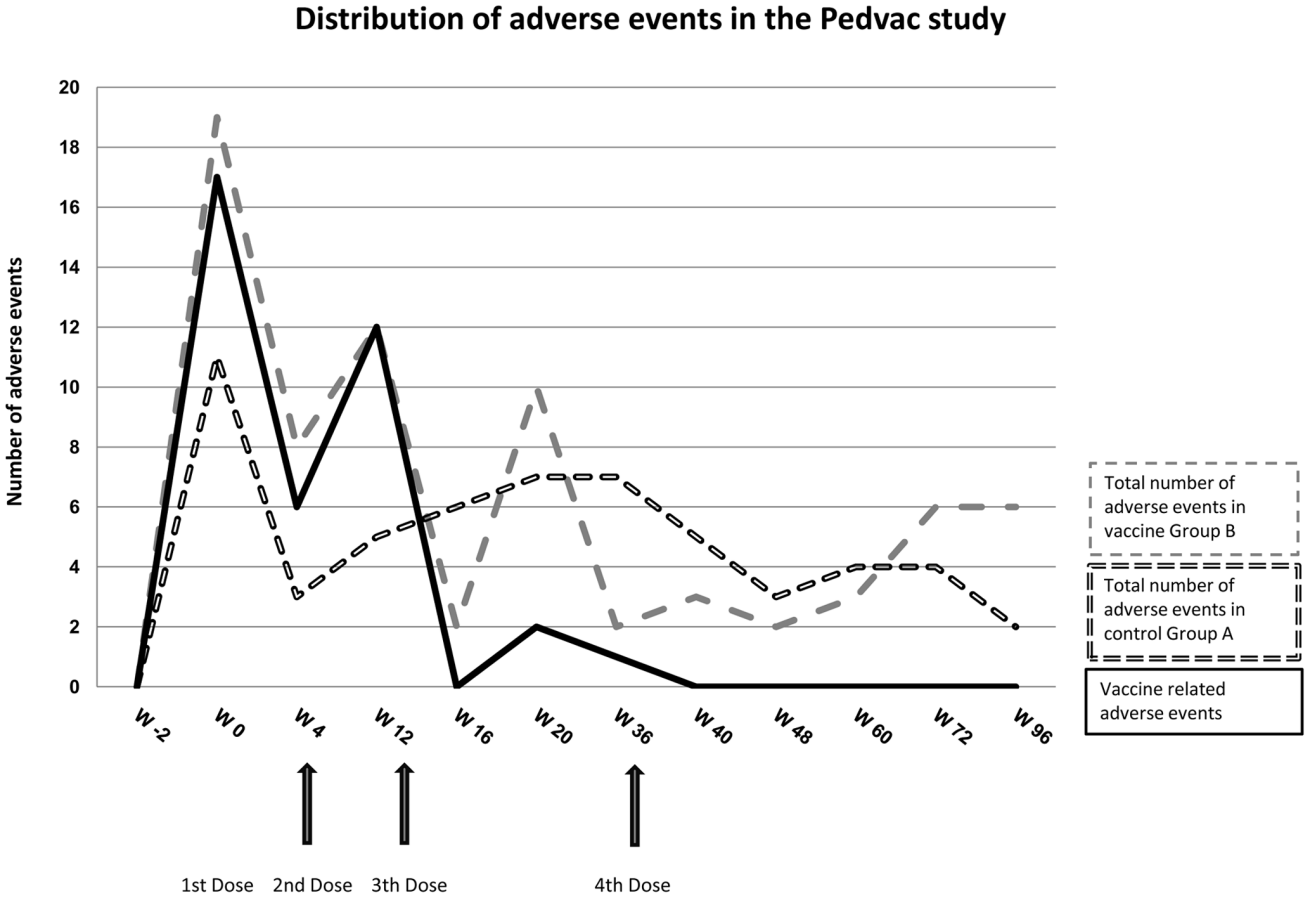
Safety Profile of the PEDVAC trial: distribution of adverse events during the 96 weeks follow-up of the study. The black dashed line shows total number of solicited and unsolicited adverse events in control Group A. The gray dashed line indicates total number of solicited and unsolicited adverse events in vaccine Group B, while the black solid line indicates all local and systemic adverse events which were vaccine related. Black arrows show vaccinations (weeks 0, 4, 12 and 36).

The vaccine and the vaccination procedures were safe and well tolerated. A total of 130 solicited and unsolicited adverse events were recorded, 73 were reported by the vaccinees and 57 by the control group. The distribution of the frequency of adverse events was similar in control Group A and vaccine Group B, with the highest frequency following the first vaccination (Figure 2). All reactions were generally self-limiting, resolving within a 72-hour period. No grade 3 or 4 events occurred and no accumulation of adverse events leading to discontinuation of the vaccination was registered. An overall of 68 solicited adverse events were recorded during the vaccination schedule in both groups (46 in vaccine Group B and 22 in control Group A). In vaccine Group B, 38 of 46 events were judged as related to the vaccination, 24 systemic and 14 local events. Local events were described as itching, swelling and pain at the injection site. Two out of these 14 local events were graded as moderate and recovered with Paracethamol therapy: a swelling reaction larger than 3 cm and an onset of moderate pain (grade 2) at the vaccine injection site. In control Group A all 22 solicited adverse events were systemic and self-limiting. An overall of 62 unsolicited adverse events (27 in vaccine Group B and 35 in control Group A) were collected and considered not related to the vaccination. Vaccinations did not influence biochemical parameters. None of the volunteers developed anti-double-stranded anti-DNA antibodies at any time point throughout the study. CD4+ T-cell counts remained stable during the study period.

Adherence to antiretroviral therapy did not significantly change during the study follow-up. Self-reported adherence to ART temporarily decreased, from 100% in both groups at baseline to 80% (2 out of 10) in the vaccinees and to 90% (1 out of 10) in the control group at week 60 (p>0,5). Periods of poor adherence to the antiretroviral regimen were decided by the patients and did not exceed a mean of 3.7 days (SD 2.3) in the control Group A vs 5.5 days (SD 1.6) in the vaccine Group B, as reported in the diaries. All patients in both groups reported that they were fully adherent to the treatment at the following week. No virological failure leading to switching of the antiretroviral regimen was observed (Table 1).

### Virological outcome

**Figure 3:**
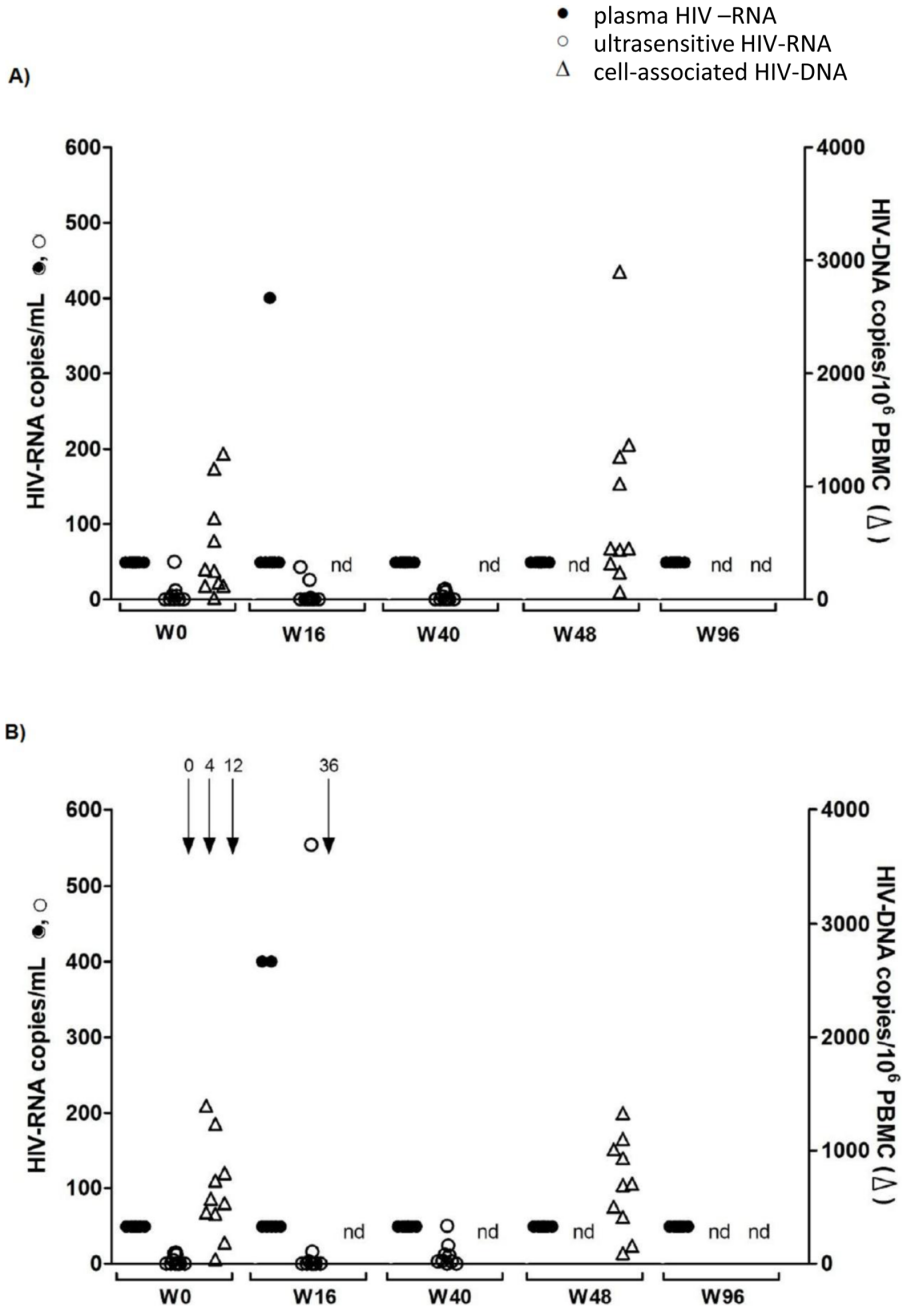
Viral dynamics during the vaccination schedule for the control Group A (n = 10) (A) and vaccine Group B (n = 10) (B). HIV-RNA with a limit of detection of 50 copies/ml is shown as black dots, HIV-RNA ultrasensitive assay with a limit of detection of 1 copy/ml plasma is shown as circles and cell associated HIV-DNA of PBMC shown as triangles indicating number of total HIV-proviral DNA copies/10^6^ PBMC. Black arrows show vaccinations (weeks 0, 4, 12 and 36).

No patient experienced virological failure. Three children (one in the control Group A, two in vaccine Group B) experienced a single viral blip of plasma HIV-RNA, which returned to under 50 copies/ml of viral RNA at the following determination. None of those three blips exceeded 1000 copies/ml of HIV-RNA, as verified also by ultrasensitive HIV-RNA determinations (Figure 3). The median cellular proviral DNA load remained stable between baseline and week 48 in both groups; control Group A: 259 copies/10^6^ PBMC (range 13–1290) vs 452 copies/10^6^ PBMC (range 66–2899); vaccine Group B: 552 copies/10^6^ PBMC (range 40–1396) vs 698 copies/10^6^ PBMC (range 93–1330). No significant differences between the two groups were observed by ultrasensitive HIV-RNA plasma viral load assays at baseline, weeks 16 and 40 or by cell-associated HIV-DNA assays at weeks 0 and 48 (Figure 3).

### Immunogenicity

**Figure 4:**
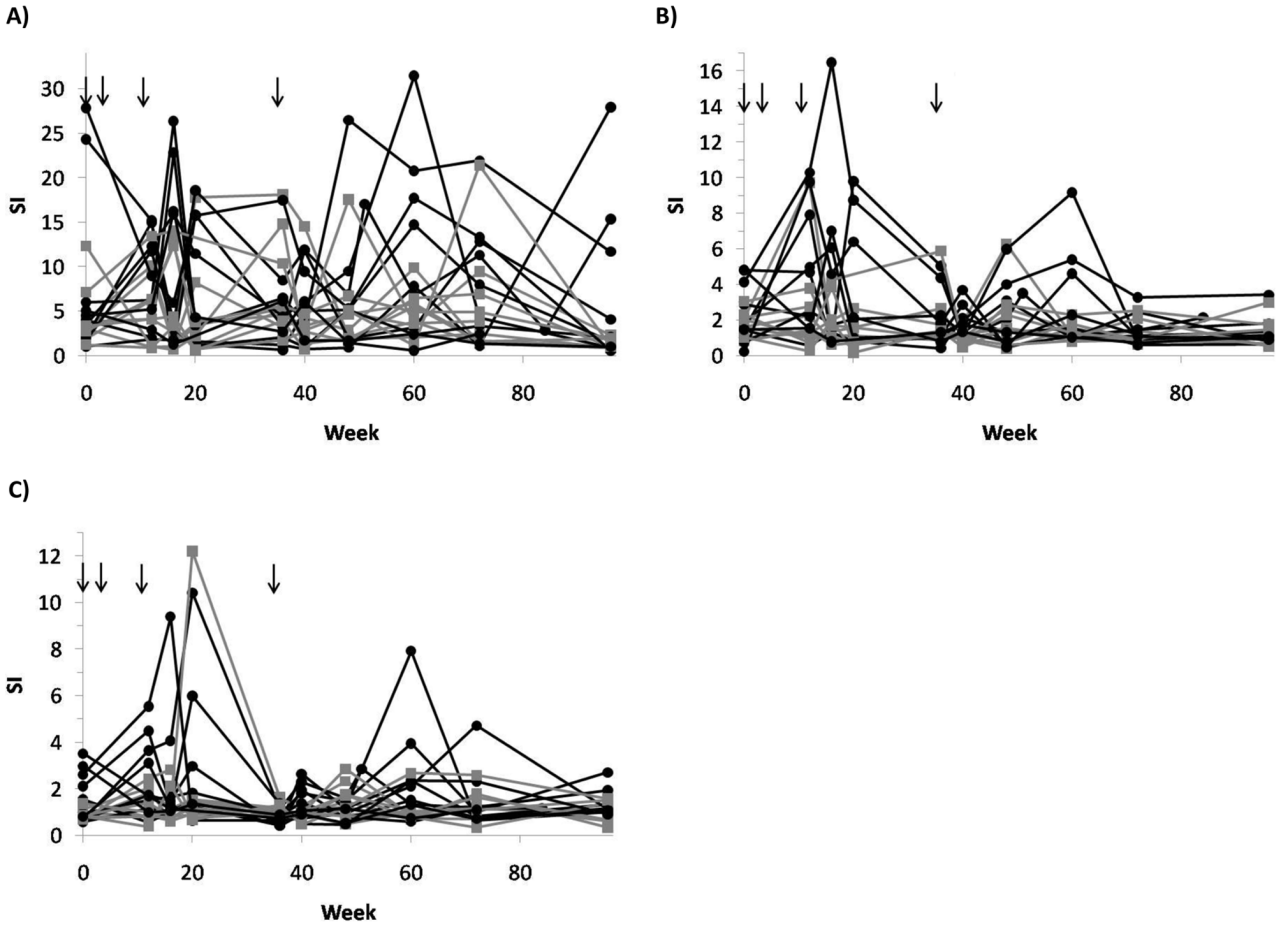
Lymphoproliferative responses are shown as Stimulation Index (SI) to virion HIV-1 MN (A), recombinant Gag p24 (B) and recombinant RT (C) viral antigens among vaccinated (n = 10; black dots and lines) and controls (n = 10; grey squares and lines). Black arrows show vaccinations (weeks 0, 4, 12 and 36).

We found a statistically significant difference between the groups as regards cellular immune responses estimated as stimulation index (SI) by the virion antigen HIV-1 MN as calculated by the area under the curve (AUC) values [18]. Figure 4 illustrates the lymphoproliferative responses to antigens HIV-1 MN, HIV p24 Gag and HIV RT, the two latter with trends to significance (Table 2). No differences were observed between the two groups regarding specific lymphoproliferative responses to antigens representing HIV Env, such as gp41 or gp160, or control CMV and Candida antigens.

**Table 2:** Cellular immune responses to HIV-1 antigens.

AUC SI^a^, mean (SD)			
Antigen	Vaccinated	Controls	p
HIV-1 MN	757.7 (387.0)	456.4 (157.6)	0.047
p24	226.7 (123.7)	148.9 (37.6)	0.073
RT	163.5 (57.1)	121.9 (39.8)	0.075
rGP160	170.6 (69.0)	125.7 (23.6)	0.067
Gp41	142.5 (40.6)	125.7 (23.6)	0.414
Candida	656.3 (352.9)	860.5 (616.5)	0.375
Cytomegalovirus	808.1 (904.2)	748.0 (629.6)	0.865

AUC of the percentage of CD3+CD4+ or CD3+CD8+ cells producing IL2 or IFN-gamma or releasing perforin, mean (SD).			
Cytokines	Vaccinated	Controls	p
CD8+ perforin	30.5 (35.8)	11.7 (11.7)	0.132
CD8+ IFN- γ	2.1 (3.7)	0.7 (0.6)	0.246
CD4+ IL-2	2.0 (1.8)	2.3 (2.3)	0.764
CD4+ IFN- γ	1.1 (0.7)	0.9 (0.4)	0.401
CD4+ IL-2 and IFN- γ	0.3 (0.3)	0.3 (0.5)	0.824

^AUC^area under the curve.

a week 0–96.

^b^week 0–60.

It can be noted that the HIV-1 MN virion antigen is thought to mainly stimulate anti-Gag reactivities. The ranked reactivities to the HIV-1 MN and Gag p24 antigens for week 16 samples, showed a correlation of r = 0.50 (p = 0.03, Spearman's test). From this we assumed that anti-Gag reactivities were the principle immune reactivities detected in the vaccinated Group B individuals.

**Figure 5:**
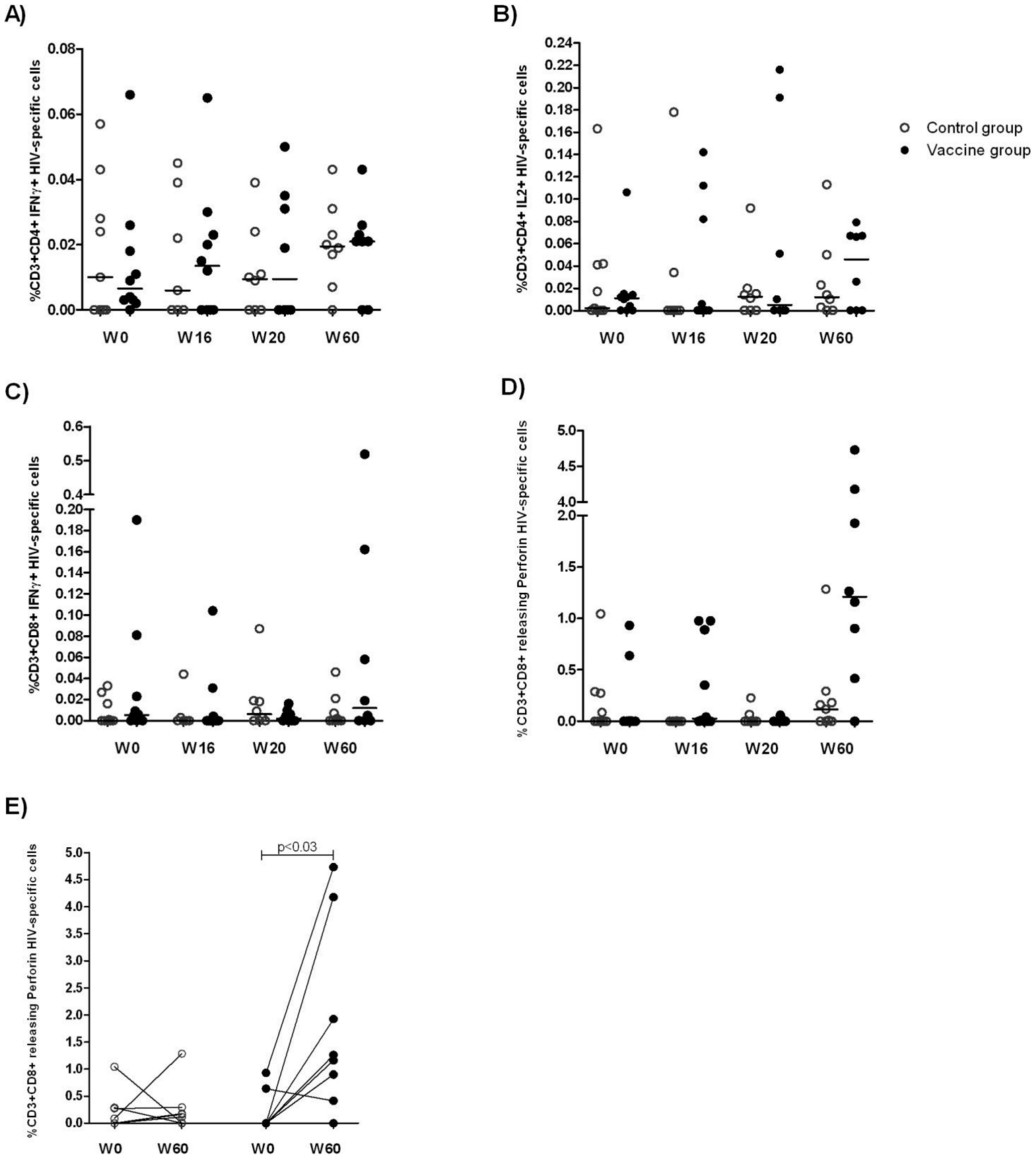
Functional profiles of between groups HIV-specific CD4+ and CD8+ T-cells analyzed by ICS at weeks 0, 16, 20 and 60. The panels show the percentage of T-cells releasing IFN-γ (A) and IL-2 (B) for CD4+ T-cells, and IFN-γ (C) and perforin (D) for CD8+ T-cells for each patient in control Group A (n = 10, circles), and vaccine Group B (n = 10, black dots). T-cell functions were analyzed after stimulation for 16 h with a pool of proteins representing the vaccine sequences. Lines represent median values. In panel (E) CD8+ cells releasing perforin at baseline week 0 and after 60 weeks in vaccine and control groups are shown. Differences within groups between baseline and week 60 levels in panel (E) are indicated for each individual, and the p-value calculated by Wilcoxon signed rank test.

The functional profile of HIV-specific CD4+ and CD8+ T-cells was then analyzed by intracellular cytokine staining (ICS) at weeks 0, 16, 20 and 60. T-cell functions were analysed by pools of proteins representing the vaccine sequences. The panel of T-cell functions analyzed included IL-2, IFN-γ and perforin production and release for both CD4+ and CD8+ T-cells (Figure 5). IFN-γ positive CD8+ lymphocytes, as well as IFN-γ positive or IL-2-positive CD4+ lymphocytes, did not differ significantly between the two groups (Figure 5 panels A–D). Table 2 summarizes p-values calculated by the AUC method for ICS determinations. Compared to baseline, the percentage of HIV-specific CD8+ lymphocytes releasing perforin in the vaccinated Group B was higher after the vaccination schedule had been completed (p = 0.031). No such increase was observed in control Group A (Figure 5 panel E).

Neutralizing antibody responses were determined in the TZMbl assay against HIV-1 strain SF162 expressed in the RT-resistant backbone 1617RT/K103N, due to the fact that the children were ART treated. Despite the modified PSV backbone, interference due to Efavirenz allowed antibody determination only for 9 children, 5 not vaccinated and 4 vaccinated. Independently of the group, most children had neutralizing antibody at baseline (ID_50_ titers ranging from 87 to 1007), which remained stable or had dropped slightly at 40 weeks of follow-up (ID_50_ titres ranging from 27 to 596). Thus, the vaccination protocol did not appear to affect antibody levels.

### Psychological impact of the PEDVAC trial

A total of 200 questionnaires covering the whole period were analyzed by the clinical psychologist after the 96 week follow-up visit. The reported quality of life was high in both groups and showed no variations during the study period. At study entry, a majority of the patients and their parents (80%) who enrolled in the vaccine group had high expectations of the trial. The possibility of being cured and discontinuing the daily intake of drugs were the main hopes reported by the vaccinees. A majority (60%) in the control group of patients wished they had been selected for the vaccine group. Comprehension of the study aims increased over time in both groups.

## Discussion

The present trial demonstrates, for the first time, the feasibility, safety and moderate immunogenicity of an HIV-DNA therapeutic vaccine in vertically HIV-infected children. While interpretations should be tempered by the relatively small study size and by the study design (HIV-DNA as both prime and boost and no withdrawal design), there are a number of significant observations that merit additional investigation.

First, HIV-DNA vaccination is a feasible and safe strategy in the pediatric population. No major adverse events were reported by the vaccine schedule. We saw no indication of increased viral load caused by vaccination. Importantly, genetic vaccination was not observed to increase the size of the viral reservoir [20]. Overall, these data confirm the safety profile of genetic vaccination reported in healthy and HIV-infected adults with the same HIV-DNA vaccine, HIVIS [7], [21], [22]

Second, genetic immunization in the context of ART is a feasible strategy since it does not negatively impact quality of life, adherence to antiretroviral drugs or viral control. In the present study, viral load was stably suppressed in both vaccinees and controls. This can be the direct result of the high adherence to ART reported in this study, due to the fact that enrolment in clinical trials increases the medication compliance and virological control [23], [24].

Third, HIV-DNA vaccination of vertically HIV-infected children, who were receiving durable effective ART, was associated with a moderate and transient appearance of HIV-1 MN (Gag) and trend to RT-specific lymphoproliferative responses. Gag specific T-cell responses have been shown to correlate with lower levels of viral load in chronically infected patients [4], [25], [26], [27], and with a better prognosis [28], [29]. Similarly, RT specific T-cell responses have been reported to be of protective value in HIV-infected individuals [30], [31]. Increased anti-RT cellular reactivities were transiently discovered in a few of the vaccinated children. This is interesting, since in healthy and infected adults vaccinated with the same vaccine, anti-RT responses were measurable in a minority of individuals. Some infected children thus might be more able to react with viral rRT proteins than infected adults, probably as a reflection of a more intact immunological status [29]. The absence of a T-cell activating adjuvant and the low endogenous antigenic stimulation due to the long-term viral suppression during the entire study is consistent with the transient and mild increase of HIV-specific lymphoproliferative immune responses observed in the present trial.

Furthermore, a significant increase in the frequency of HIV-specific CD8+ lymphocytes releasing perforin was observed after HIV-DNA vaccination at week 60 when compared to baseline values. However, we cannot distinguish if these effects result from the induction of true *de novo* responses or derive from preexisting antigen-experienced T-cell responses. In HIV-infected children with complete viral suppression, the HIV-specific CD8+ cellular responses decrease below detection, showing that active viral replication is required to maintain such responses at detectable levels [32]. The observation that the majority of the HIV-specific CD8+ cellular responses at baseline in our study were below detection is in line with these data. Therefore, the persistently low virological burden observed in our cohort suggests that the HIV-DNA vaccination can increase the frequency of CD8+ lymphocytes releasing perforin. However, it is likely according to previous data in vertically HIV-infected children [32], that these cells do not remain fully functional in the absence of the continued antigenic stimulation provided by vaccination or transient viremia.

Fourth, boosting vaccination with HIV-DNA after HIV-DNA priming was not a fully effective strategy. The HIV-DNA vaccine contains full genetic information for Gag, RT and Env proteins, together representing subtypes HIV-1 A, B and C. This theoretically best covers both the viral variation and the many polymorphic genes/gene products of HLA classes I and II alleles, but perhaps at the cost of strong peptide-focussed responses. A heterogeneous approach in which the boost consisted of recombinant vaccinia- or adeno-based HIV genes has led to broad and strong cellular immunities followed by antibody induction in healthy adults [7], [22], [33], [34], [35]. A DNA vaccine carrying CpG sequences that engage Toll-like receptor 9 (TLR 9), which in turn redirects the immune response from Th2 to Th1 reactivity [36], can be another feasible and innovative strategy to enhance cellular immunogenicity of DNA vaccines. Other new experimental strategies, as in vivo electroporation or cytokine genes, might enhance the potency of DNA immunizations to match immune responses induced by heterologous prime-boost immunizations [10], [37]. It was however not possible to design similar prime-boost or experimental immunization schedules in the childhood population without prior demonstration of safety of genetic HIV vaccination. The fact that no novel neutralizing antibody responses were elicited is disappointing, but consistent with the performance of other stand-alone DNA vaccines delivered to date [37].

Overall, our data pave the way to explore immunotherapeutic approaches in the pediatric HIV population, particularly aiming at increasing the strength and durability of anti-viral responses. The role of HIV therapeutic vaccines in childhood is debated due to the efficacy of antiretroviral therapy in maintaining long-term virus suppression. Antiviral therapy and therapeutic vaccination have mostly been perceived as alternative intervention strategies, particularly by the patients and their families. The major goal of a therapeutic vaccination thus far, has been the induction of cellular immune responses that control viral replication, thus allowing the interruption of antiretroviral therapy. However, it is unrealistic to think that therapeutic vaccination will stimulate HIV-specific T-cell responses to achieve full and persistent HIV-viral suppression as efficiently as a combination of antiretroviral therapies. Therapeutic vaccination thus should target those patients who present high risk factors to develop virological failure and viral resistant strains, with the final aim to reduce viral rebound in less adherent patients. The largest pediatric epidemiological study in Europe recently reported that 12% of HIV infected children had triple-class virological failure after 5 years on ART, and about a fifth had such treatment failure by 8 years on ART [2]. Therefore, this should be the pediatric population to target with an improved therapeutic vaccine. Such a vaccine would have to show a further decrease below the set point induced by an anti-retroviral treatment in order to qualify for a complementary approach.

A longer follow-up of children enrolled in this study would better clarify the impact of the present vaccine on the long term viral control. In parallel, larger trials are needed to analyze the short- and long-term efficacies of therapeutic vaccination in reducing the rate of virological failures in high risk pediatric populations.

## Supporting Information

Checklist S1 **CONSORT Checklist.**
(DOC)

Protocol S1 **Protocol for trial.**
(PDF)
