# Supplementary material for: Therapeutic DNA Vaccination of Vertically HIV-Infected Children: Report of the First Pediatric Randomised Trial (PEDVAC)
Source: PLoS One. 2013 Nov 28;8(11):e79957. doi: 10.1371/journal.pone.0079957 (PMC3842924; doi:10.1371/journal.pone.0079957)
Supplement: Protocol S1 — Protocol for trial. (PDF) [file pone.0079957.s002.pdf]

PEDVAC Trial :

Therapeutic vaccination with HIV DNA plasmids in vertically HIV infected children under HAART

Dipartimento di Pediatria

Unità Operativa di Immuno-Infettivologia

Ospedale Pediatrico "Bambino Gesù"

P.zza S.Onofrio 4

00165 Roma

Italia

Principal Investigator	*Sponsor*
Prof. Paolo Rossi	*Swedish Institute for Infectious Disease Control (SMI)*

Contatti

Prof. Paolo Rossi
Phone + 39 6 68592190
Fax + 39 6 68592508
rossi@opbg.net

Trial Coordinating Committee

Paolo Rossi

Stefania Bernardi

Paolo Palma

Britta Wahren

Scientific Advisory Board

Gianfranco Bottazzo

Eric Sandstrom

Massimo Andreoni

Viviana Moschese

Background

In 2008, the devastating impact of the HIV/AIDS pandemic continues unabated. UNAIDS estimates that around 4 million people were newly infected with HIV in the year 2007 and that 420.000 of these infections occurred in those aged less than 15 years, most living in sub-Saharan Africa. Paediatric HIV infections are acquired both vertically and horizontally and HIV vaccines might be developed to combat both modes of transmission.

The development of an effective HIV vaccine is a major global health priority. In younger age groups, vaccines are greatly needed to prevent horizontal HIV transmission in adolescents and acquisition of HIV infection from breast-feeding in infants and to enable prolonged treatment interruptions in HIV-infected ones. To date, development of vaccine candidates has been of limited success, demanding innovative approaches to vaccine technology. Inactivated and live attenuated vaccine technology, so successful in vaccinology of other pathogens are considered too risky, since HIV infection may occur. Subunit vaccines have been ineffective, failing to elicit neutralizing antibody responses. Therefore sterilizing immunity, which is thought to be critical for prevention of infection, seems an unlikely prospect. Current promising DNA and vector-based vaccine candidates in or about to enter phase I trials are designed to induce cellular immune responses that prevent persistent infection or disease development rather than acquisition of infection. Means are also being developed to induce protective antibodies by genetic vaccination.

DNA vaccination at birth can prime both CTL and antibody responses in animals even when the recipient carries maternal antibody, providing long-term protection in experimental animals. In contrast to the strong IgG2a skewing of the humoral immune response after conventional vaccination in neonates, DNA vaccines expressing the same antigen may induce similar IgG1 and IgG2a levels in neonate and adult animals. Therefore, DNA technology holds promise for use as neonatal HIV-1 vaccines. No HIV-1 DNA vaccine has been tested thus far in children. In view of a recent failure of an HIV vaccine based on a viral vector (Sekaly 2008), our DNA vaccine appears suitable since it does not contain any vector.

An effective vaccine administered in the neonatal period would prevent HIV infections due to *in utero* or intrapartum exposure. However, breast-feeding by infected mothers carries a risk of vertical transmission especially in developing countries where formula milk feeding is either unavailable or unaffordable and may themselves be associated with a high risk of morbidity. A vaccine that induced an effective immune response in the newborn could

reduce this transmission and enable prolonged treatment interruptions. Horizontal transmission is predominantly sexual, and in sub-Saharan African countries sexual debut can be as early as at 10 years of age, with much trans-generational sex, putting the pre-adolescent at risk for HIV acquisition. Adolescents in the developed world are also at high risk of acquiring HIV-1, with 20 000 new infections in 13–24-year olds annually in the United States alone. Children and adolescents are therefore important targets for preventative or therapeutic HIV vaccine clinical trials. To date such trials have been conducted almost exclusively in adults. None of the preventative or therapeutic HIV vaccine trials have included adolescents and only two completed trials targeted HIV-1 exposed neonates (PACTG 326 and PACTG 230) (Lamert 2004, Jaspen 2006).

In order to license a vaccine for use in children, data should exist on the safety and immunogenicity or efficacy of the vaccine in this age group. Development of vaccines in children is facilitated where there are specific correlates of immunity. For example, trials in adults demonstrated that hepatitis B surface antibody (HepBsAb) titres of greater than 10 IU/l conferred protection against hepatitis B infection. The use of this surrogate marker enabled testing and approval of the recombinant Hepatitis B vaccine for neonates. However, in the absence of correlates of immunity to HIV-1 infection the approval of a vaccine for children will require efficacy data and will thus take longer. Furthermore, there are physiological and immunological differences between children and adults that may change the safety and efficacy of developed vaccines. However there is also a compelling argument that such vaccines are needed and that a moral imperative exists to develop and test candidate vaccines in paediatric age.

Immunotherapy which would enable prolonged treatment interruptions with immunologically mediated viral control would be very valuable and would represent the next step for a further preventive vaccine clinical trial.

The purpose of this study is primarily to highlight the safety, feasibility and immunogenicity of HIV DNA vaccine in a cohort of HIV infected children highly characterized for their ability to respond to HIV antigens (Pensieroso AIDS 2006, Palma AIDS 2007). Thus, this is an ideal group of young persons to test an HIV vaccine. The HIV vaccine aims to induce new immune responses which in turn may allow a reduction in therapeutical burden. A reduction of the present ART regimen which may include toxic drugs and has to be taken daily during the whole life would be a major advancement. This will be of great significance particularly in infants and children where lifelong antiretroviral therapy may be associated with severe and unpredictable side effects. From two phase 1 studies in healthy adults and one study in HIV infected adults with this vaccine, we have noted only minor local side effects from the HIV

DNA part of the vaccine schedule. Therefore we expect no major side effects from the HIV DNA vaccine.

Previous therapeutic HIV vaccine studies by this group

The Swedish group has been working with therapeutic immunizations since the late 1980's with phase 1 to 3 trials and is currently involved in two major projects funded by the European union on other aspects of HIV immunization. This study represents another close collaboration between pre-clinical and clinical science.

A number of studies have been performed with the intention to evaluate immunotherapy in HIV infection at South Hospital, Sweden. The first studies were initiated as phase 1 studies with HIV outer membrane protein (baculovirus produced gp160). The Swedish group demonstrated a positive effects on T cell proliferation and CD4 counts. Therefore a randomised inter-Nordic multi-centre double blind placebo-controlled trial with 835 HIV-1 infected individuals was performed with the intention to establish possible clinical benefit of rgp160 immunization. HIV-1-specific T cell immune reactivity was induced in all vaccine recipients studied and they had a modest CD4 cell count increase. Significantly fewer deaths were observed among the vaccine recipients compared with the placebo patients at two years. At the end of the study at three years this effect was no longer evident (Sandstrom and Wahren 1999).

These studies have been continued with HIV protein, vaccinia vector and DNA immunogens (plasmid DNA carrying the HIV regulatory genes nef, rev and tat) with and without combination HAART (Hejdeman et al 2004). At present an immunotherapeutic study is ongoing in Stockholm where the HIVIS DNA vaccine is delivered by the skin method Dermavir. DNA vaccines are of interest because of their ability to mimic the effects of live attenuated vaccines and to induce both humoral and cellular immune responses. A corresponding breath in the immune response is not induced by protein vaccines. In summary the previous studies have shown that new memory cells can be induced by vaccination in the chronic phase of HIV-1 infection. These responses may last for several years irrespective of HAART as long as the CD4 T cell count is adequately controlled.

HIVIS, HIV Immunogenicity Study in healthy adults.

A previous phase 1 study of a preventive HIV plasmid DNA vaccine has been concluded at the South Hospital of Stockholm, comprising 40 healthy volunteers as a safety trial. In this human study HIVIS DNA was used as a prime followed by MVA boost vaccination. Safety and immunogenicty data are collected in Stockholm, Sweden. This was followed by a

second phase 1 study, which has started in Tanzania. The Regional ethics committees and the Medical Products Agencies have approved this trial and the immunogens. The same immunogens and schedules are used in this second study, which has recruited 60 individuals of whom 40 in the SHr blindest trial with no safety concers. facilitating regulatory approval and also comparison of immunological responses.

The Swedish HIV DNA prophylactic study has enrolled 40 healthy volunteers, 33 males, mean age 32.6 years and 7 females of non-childbearing potential, mean age 54.7 years. They were treated with three doses of the multiclade/ multigene HIV-DNA vaccine (HIVIS) and 1 single dose of modified vaccinia virus Ankara (MVA) and followed for over 1 year .

The primary objective of the study was to evaluate the safety of three dose levels of HIVIS in this study population:

HIVIS study

Arm	***Number***	***Mode of DNA Immunization***	***Adjuvant***	***Ampoule 1 (env) Left arm***	***Ampoule 2 (gag, Rtmut) Right arm***
A	*10*	*Id*<br>*Bioject*	*none*	3 injections of 100ul<br>*(tot 0.6mg DNA)*	*2 injections of 100ul*<br>*(tot 0.4mg DNA)*
B	*10*	*Im*<br>*Bioject*	*none*	*1.0 ml*<br>*(tot 2.0 mg DNA)*	*0.9 ml*<br>*(tot 1.8 mg DNA)*
C	*10*	*Id*<br>*Bioject*	*GMCSF(Leukine® (sargramostim) Berlex, 0.5 ml - 150ug)*<br>*Sc with needle.*<br>*150 mcg under id injection site left arm*	3 injections of 100ul<br>*(tot 0.6mg DNA)*	*2 injections of 100ul*<br>*(tot 0.4mg DNA)*
D	*10*	*Im*<br>*Bioject*	*GMCSF (Leukine® (sargramostim) Berlex, 0.5 ml - 150ug)*<br>*Im Bioject.*<br>*150 mcg at injection site left arm*	*0.6 ml*<br>*(tot 1.2 mg DNA)*	*0.45 ml*<br>*(tot 0.9 mg DNA)*

The volunteers were randomised to the different treatment arms by the selection of a consecutive sealed numbered envelope containing a slip labelled A to D prepared by the study monitor by a block randomisation procedure.

The DNA vaccine consisted of plasmids encoding HIV env A, env B, env C and rev in one ampoule and of plasmids encoding gag A, gag B and RT in another ampoule.Group A individuals were immunized intradermally, (id), group B intramuscularly, (im) , Group C id as group A plus GMCSF subcutaneously, sc, in the left arm and group D im as group B but half the DNA dose plus GMCSF im in the left arm.

All volunteers obtained the pool of env A, B and C and RevB plasmids in the left arm and the pool of gag A and B and Rtmut B in the right arm.

All injections except the sc GMCSF injection were given with the Bioject device, Bioject Medical Technologies, Inc., Tualatin, OR, USA.

Id injections were divided in three 0.1 ml injections in the left arm and two 0.1ml injections in the right arm.

Im injections were divided in one 1ml injection in the left arm and 0.9ml in the right arm, group B or 0.60 and 0.45 in the left and right arm respectively, group D.

Immunizations were given at day O, day 28 +/- 2 days and day 94+/-7 days. Volunteers remained 30 minutes in the clinic, were contacted after 2 days by telephone and filled in a diary for 7 days (including standardized measurement of body temperature) after each injection.

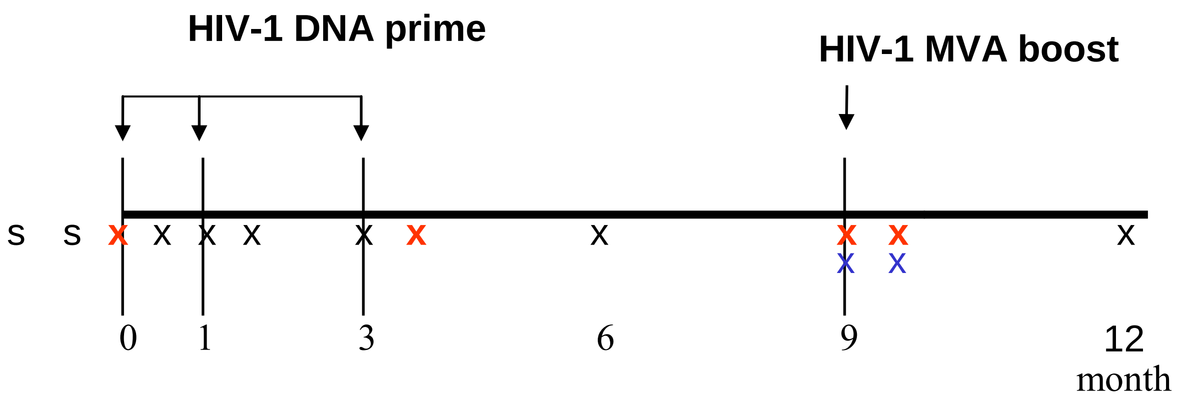

S = screening

X = visit with fresh Elispot IFN-γ assay

X = visit with lymphoproliferative assay

X = visit with additional safety labs

Samples for safety analysis were obtained 4-8 weeks before, at and 2 weeks after each immunization and 3 months after the last immunization. In addition active hepatitis B and C, and syphilis were excluded at the first screening visit and tests for lymphocyte subsets, ANA, anti – ds DNA and CPK were performed. The safety labs included haemoglobin, white blood cell count, neutrophils, lymphocytes, platelets, fasting glucose, bilirubin, AST, ALT, ALP, creatinine and a complete urinalysis.

To date, all cohorts completed the treatment phase of the study. Two patients discontinued the study autonomously due to toxicity (grade 2). A significant increase(P<0,00001) of HIV-specific lymphoproliferative response was detected in 37 out of 38 patients at the end of the study. A lower lymphoproliferative response was observed in ARM D using vaccine plus GM-CSF intramuscularly delivered (Figure 1)

Figure1: Lymphoproliferative response to HIV-AT2 inactivated virus in HIVIS-study at the end of the study.

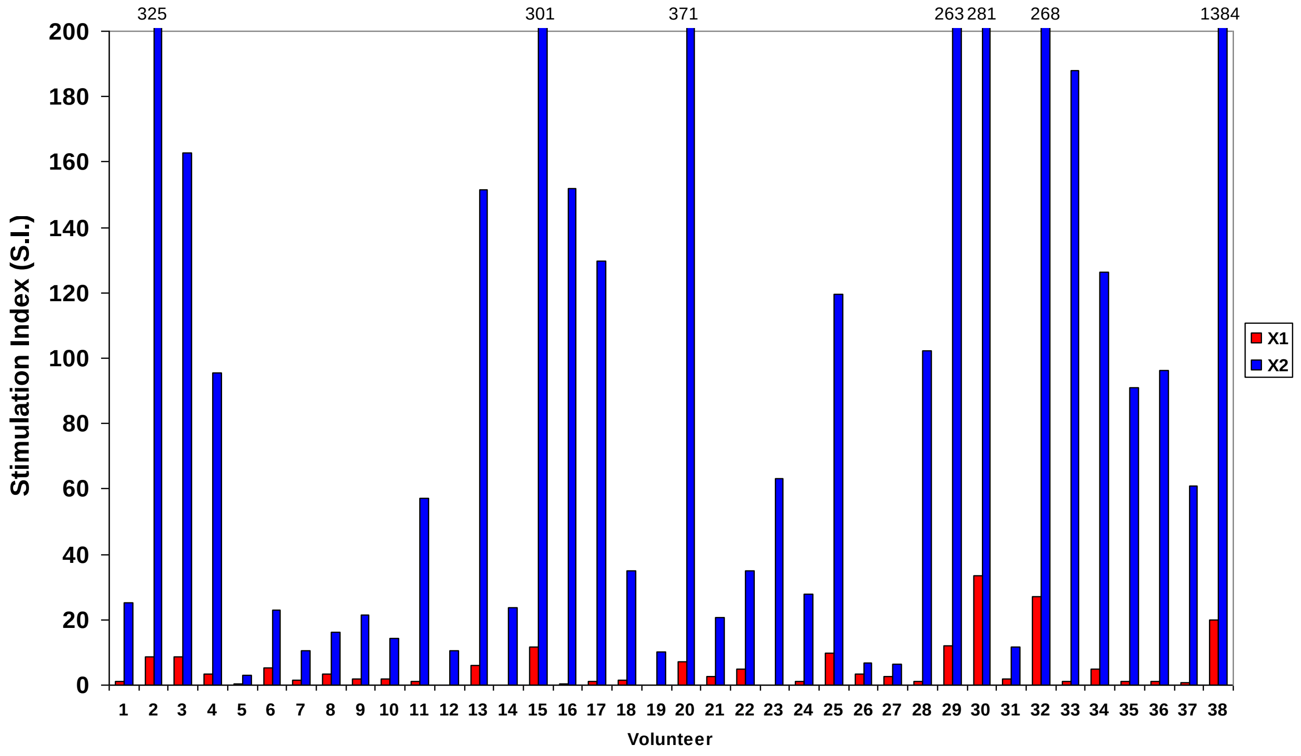

Cytotoxic response measured by ELISPOT for IFN-γ gave positive results in 33 out of 38 patients at the end of the study. Interestingly, younger persons react with higher elispot reactivity (Figure 2), and total spot reactivity for IFN- γ resulted in higher numbersat the end of the study in ARM B, using DNA vaccine intramuscularly delivered without GM-CSF (Figure 3) .

Figure 2: Cd8+T-cell Elispot for IFN-gamma detected at different ages shows a higher reactivity in younger patients.

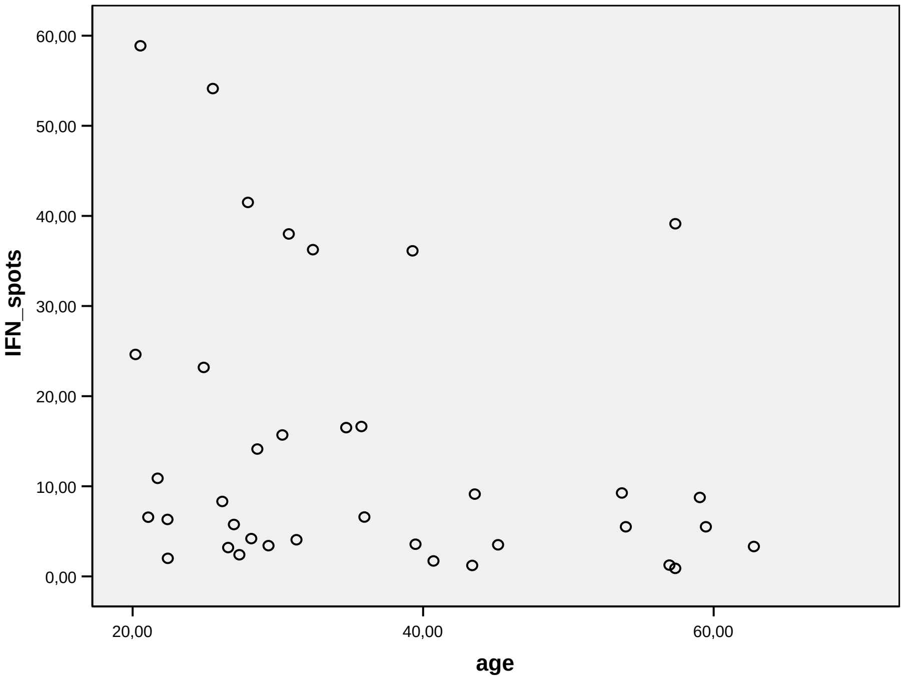

Figure 3. Elispot for IFN-gamma in different arms the end of the study .

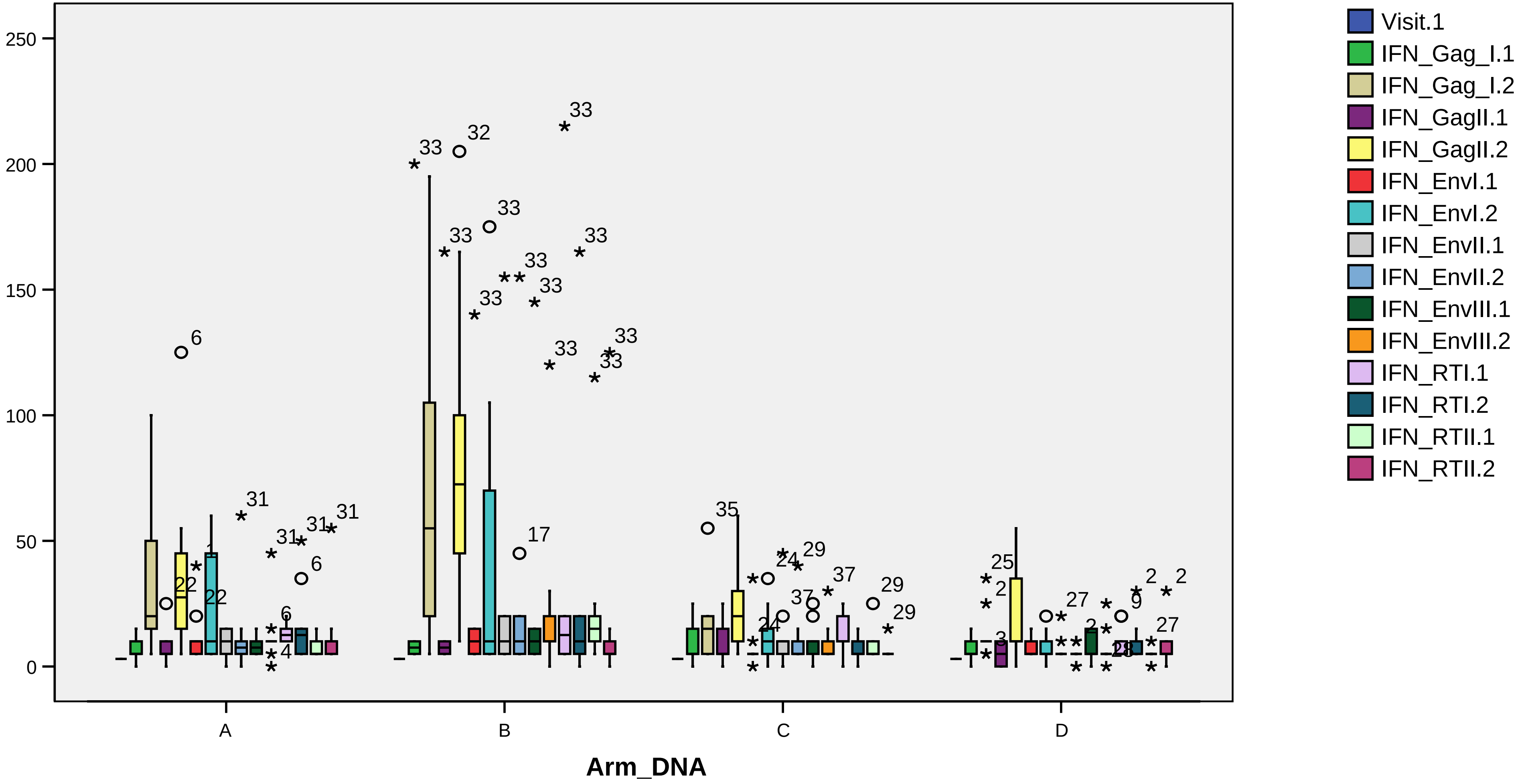

Clinical tolerability

Over all the vaccine was well tolerated. A total of 360 adverse events were recorded in the six visits from the first injection to 2 months after the last, of which 274 were possibly or probably related to the vaccination. More than half (155) were local reactions, 43 were 'influenza' like symptoms, 40 were feeling tired. Twenty-three were grade 2. Most of the side effects reported were related to the use of GM-CSF (ARMS C,D). The events that led to the termination of one volunteer in group C were recorded as grade 2 by the study personnel and 3 by the volunteer and were characterized by headache, chills, nausea and excessive tiredness requiring her to stay home from work for 2 days. There were no local reactions. The reactions to the immunizations did not change over time in any group. There were 5 grade 2 'influenza' events reported by the 7 women than among 4 in 33 men.. All other symptoms were similar by gender.

Safety labs

There was no influence of the vaccinations on haemoglobin, WBC, neutrophils, lymphocytes, platelets, ASAT, ALAT, alkaline phosphatase, bilirubin, creatinine or fasting blood glucose.

Gender

According to EU rules fertile women can not be included in phase 1 studies unless reproductive toxicology studies have been performed. The study came to include some menopausal women and thus women were older than the men in the study. Since the primary aim is safety this was acceptable although there is a risk of lower immune responses.

PEDVAC study: a phase II clinical trial on feasibility and immunogenicity of HIVIS DNA vaccine in HIV-infected children.

The primary objective is:

1. To document the feasibility and immunogenicity of HIV plasmid DNA immunization intramuscularly delivered to HIV vertically infected children.

The secondary objectives are:

1. To investigate safety of DNA plasmids carrying HIV genes intramascular delivered in HIV vertically infected children under effective HAART treatment.
2. To correlate the viral load and CD4+ counts after the DNA vaccination to immune responses
3. To characterize the new HIV specific immune responses by CellElisa, T-cell proliferation and antibody reactivity.
4. To study the effect on CD4 counts in the two groups.

Study subjects

20 HIV-1 vertically infected children, on stable antiretroviral regimen for at least 6 months with less than 50 copies/ml of plasma HIV-1 RNA at two determinations over 12 months, will be randomised to 2 groups. They should have stable CD4 value above 400 cells/mm^3 or 25% in the last 12 months of follow-up.

Study design

Phase II open label controlled, randomised, laboratory blinded trial, evaluating safety and immunogenicity of HIVIS-DNA vaccine in a cohort of 20 children randomised in 2 groups as follows:

- Arm A: a group of 10 children who will continue previous HAART regimen.
- Arm B: a group of 10 children who will be immunized with HIVIS-DNA vaccine in addition to previous HAART regimen, in accordance with the following schedule:

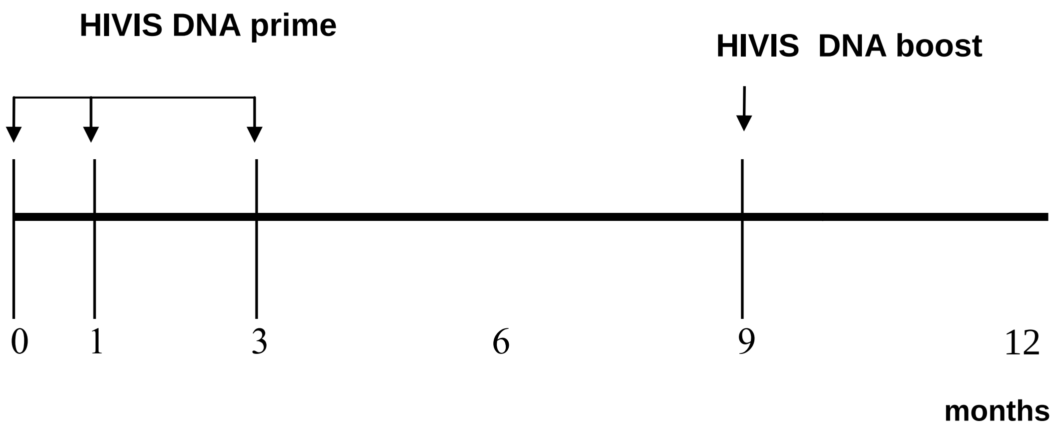

At each immunization every patients in Arm B, will received 2 intramuscular injections in two different sites (i.e. left and right arm), for a total of 8 intramuscular injections.

Vaccine and formulation

Two preparations of DNA will be prepared using the HIVIS plasmids.

1. DNA plasmids (2mg/ml) with HIV env A, B, C and Rev B. (Ampoule1 0.7 ml pKCMV(rev,envA,envB,envC)
2. DNA plasmids (2mg/ml) with HIV gag A, B and RT mut B. (Ampoule 2 0.5 ml pKCMV(gagA,gagB,RTmut))

The vaccine has been manufactured in accordance with GMP standards by Vecura (Karolinska University Hospital/Huddinge) and previously approved by the Medical Products Agency (Läkemedelsverket) for the prophylactic HIV vaccine study of 40 healthy adults in Sweden.

For intramuscular injection each child will receive 4 ampoules (2 of no.1 and 2 of no.2), in total 3.8 mg DNA. **This corresponds to arm B in the Stockholm HIVIS protocol** (therefore also to be called arm B in the PEDVAC protocol).

The study site

The trial will be performed at the Clinical Trial Center of the Children's Hospital "Bambino Gesù" where several phase I /II trials have previously been performed in the field of treatment of paediatric HIV.

Inclusion criteria

1. Aged between 4 and 16 years
2. Partecipants to the study will be cautioned of the unknown hazard to a foetus and advised to not have sexual relations for the duration of the study.
3. HIV infection detected by two serological and HIV plasma RNA tests
4. On HAART for at least 6 months with less than 50 copies/ml of plasma HIV-1 RNA at two determinations over 3 months
5. Stable CD4 value above 400 cells/mm^3 or 25% in the last 12 months of follow-up
6. Viral isolate pre –HAART available is preferable but not mandatory
7. Able to give informed consent
8. Availability for follow-up for planned duration of the study

Exclusion criteria

1. Patients with ongoing infection(s) other than HIV
2. Treatment with steroids, systemic or local at the immunizing sites, one month before or during the trial
3. Patients with signs of autoimmune diseases
4. Patients with creatinine > 2mg/dl, Hb < 12g/dl, leukocytes < 3,000μl, platelets <150,000/μl
5. Patients on any immune modulating or investigational drug
6. Anamnestic allergy to kanamycin or plasmid gene products

Endpoints

1. Any grade 3 or above toxicity possibly or probably related to the immunization.
2. Virological failure defined as 2 determinations over 1000 copies/ml consecutively.
3. Immunologic failure defined as progression in the CDC classification status.
4. Clinical failure defined as progression in the CDC classification status.

Recruitment

Participants will be recruited from the patients seen at Department of Paediatrics, Unit of Immune and Infectious Diseases, Children Hospital “Bambino Gesù” in Rome. Individuals known to conform to the study criteria will be informed about the trial either through a letter with the patient information or through their physician and invited to a pre-screening visit, visit 1, to be better informed. Informed consent will be signed at this visit and the patient given a consecutive study number eg. PEDVAC 73309-xxxx. Eligible patients will be notified of the study by their primary physician in conjunction with a regular visit when safety and HIV monitoring studies are performed in the routine and given written information of the trial.

Participants will then notify the study nurse of their interest and given full trial information, sign an informed consent form and the patient given a consecutive study number eg. PEDVAC 73309-xxxx, if still interested. Thereafter, at visit 2 Hepatitis B and C, CMV and ANA; anti ds DNA, and safety labs [HB, WBC, differential counts, platelets, ASAT, ALAT, ALP, creatinine and urinalaysis] will be analysed.

No more than 3 patients a week will be recruited over the first 4 weeks.

Participants will be warned not to father any children for the duration of the trial.

Any reimbursement is provided to the participants of the study.

Randomisation

The randomization schedule will be prepared by the statisticians at the Clinical Trial Center of "Bambino Gesù" in Rome. The randomization list will be performed by Electronic Software available at RANDOM.ORG. A list will be prepared consisting of 2 blocks each containing 10 children assigned to Arms A or B A list will be prepared consisting of 2 blocks each containing 10 children assigned to Arms A or B. Envelopes, numbered corresponding to the list, containing the assignement will be kept at the pharmacy. After a patient has signed the informed consent at visit 1 the study nurse will notify the pharmacy using Pharmacy form 1, where the next consecutive envelope will be opened and the form returned to the clinic indicating if HIV-DNA vaccine should be used or not. A copy, Pharmacy form 2, will be kept under lock at the pharmacy containing all the information in the envelope. Subsequently vaccine or single HAART will be requested using Pharmacy form 3.

Safety samples will be sent to the laboratories under the personal numbers used for purposes of clinical care.

Study samples will be sent to the participating laboratories under the study numbers eg. PEDVAC 73309-xxxx.

The investigator will keep a log of patients screened for the study, but not included, and will give the reason for exclusion.

Analyses

Visit

At each evaluation, patients will underwent to a complete medical history and a physical examination that included weight, height and blood pressure measurements.

Blood Chemistry

Blood chemistry will performed at baseline andat weeks -2, 0, 10, 20, 36, 40, 48, 60, 72 and 96. Laboratory evaluations obtained after at least 8 h of fasting included complete , blood chemistries (glucose, blood urinary nitrogen,creatine, electrolytes, total proteins, albumin,

total and direct bilirubin, aspartate transaminase, lactate dehydrogenase, creatine kinase, amylase, lipase), metabolic evaluations [total cholesterol, high density lipoproteins (HDL), low density lipoproteins (LDL), triglycerides,insulin and lactic acidaemia] and urine analisys.

Cell counts:

Cell counts will be performed at week -2, 0, 10, 20, 36, 40, 48, 60, 72 and 96 and include:

- Blood count that will performed at baseline and at
- T lymphocytes subsets will be determined by standard flow cytometric procedures using conjugated monoclonal antibodies to CD3, CD4, CD8, CD19, CD16, and CD56at week -2, 0, 10, 20, 36, 40, 60, 72 and 9.6Optionally other white blood cell markers will be analysed to reveal immunological memory (CD45, ki67 and others).

Virology:

- HIV-RNA load will be quantified during the study using HIV monitor (Roche Diagnostic System, Branchburg, NJ) at week -2, 0, 16, 20, , 40, 48, 60, 72, 84 and 96
- DNA load will be quantified at the same time.
- DNA genotyping will performed at week 0 and 60
- Samples will be stored for optional resistance testing.

Sierology:

Changes in titers of neutralizing antibodies will be studied by ELISA and type and subclass determined.

Saliva and urine will be collected for determination of specific IgA.

Anti nuclear antibody (ANA) and anti-dsDNA antibodies may be determined on frozen samples if indicated by suspected auto-immunity.

Lymphoproliferative response

Cells will be analysed fresh directly but also stored at baseline and continuosly. Immunogenicity will be tested by in vitro analysis of lymphoproliferative response to mitogens (PHA), recall antigens (candida and tetanous) and HIV antigens: the whole inactivated HIV-virus, gp160, gp120, p17, p24 .

CellElisa

Epitope mapping of the T-cell response will be tested through CellElisa technique. A new micromethod (LiPira 2007; 2008) will be applied using as few as 10 x 10^3 PBMC per tested antigen or peptide, instead of 400 x 10^3 PBMC required by conventional assays like lymphoproliferation, ELISPOT or intracytoplasmic cytokine staining, to test cellular responses

to peptides representing the vaccine genes. The assay based on re-thinking of established techniques like ELISA and ELISPOT in array format, allowing assessment of broad peptide panels with limited blood samples. This makes the assay suitable for paediatric applications and permits simultaneous assessment of different cytokines secreted by antigen activated T cells. The miniaturized assay performed in 384 or 1536 well plates is highly automated. In this way few lymphocytes from the infected and vaccinated child can give rise to a large amount of information on the status and changes of antigen specificity and innate immunity during immunization.

HLA typing

HLA Class I-typing will be performed at week 48.

Immune activation (optional)

The degree of expression of molecules involved in apoptosis (Fas and FasL) and immunological activation (CD27, CD28 and IL-7R) and proliferation (Cyclin A2) will be measured on subpopulations of T and B cells in blood.

Strain characterization

Viral strain characterization will be performed at Visit 2 week -2.

Biobank.

All samples will be stored under study code at Children Hospital “Bambino Gesù”.

Individual experience and quality of life (QoL)

Health related quality of life and individual experience will be measured by the PENTA (Paediatric European Network for treatment of AIDS) questionnaire and by a specific questionnaire developed for this study, adapted from WHOQOL-HIV. The individual experience will be further explored by qualitative analysis of interviews with the participants.

Flowchart

**Week**	-4	-2	0	4	12	16	20	36	40	48	60	72	84	96
VISIT	**1**	**2**	**3**	**4**	**5**	**6**	**7**	**8**	**9**	**10**	**11**	**12**	**13**	**14**
Enrolment	x													
Vaccination			**V**	**V**	**V**			**V**						
Blood chemistry		x	x		x	X	x	x	x	x	x	x	X	X
Cell counts		x	x		X	X	x	(x)		x	x	(x)	X	X
RNA load<br>DNA load		x<br>x	x<br>x			x<br>x	x<br>x		x<br>x	x<br>x	x<br>x	x<br>x	x	x<br>x
Resistence			x		(x)				(x)		X			(x)
Sierology		x	X		X	X	(x)	x	x	x	X	(x)		X
Lymp.Assay		x	X		(x)	X	x		x		X	x		X
CellElisa		x	X		X	X	x		x		X	x		x
Imm. Activ		x	X		X	X	x		x		X	x		x
HIV- Clade characterization		x												
HLA typing										x	x			
QoL	x		X	x	X	X	x	x	x	x	x	x	x	x
Blood volume, ml		14ml	14ml		14ml	14ml	4ml	14ml	14ml	4ml	14ml	4ml	4ml	14ml
Extra blood volume		10ml	10ml		10ml	10ml		10ml	10ml		10ml			10ml

(shaded)	Regular visits
**V**	Immunization
(striped)	Additional study visits
x	Study samples, (x) stored for later analysis

Clinical follow-up

After giving informed consent participants will be given a medical examination and inclusion and exclusion criteria will be reviewed at the first study visit. They will continue the previous antiretroviral treatment started before the enrolment in accordance to their primary physician. At the enrolment in the trial (week-4) a written informed consent will be obtained from each patient's legal guardian before study entry , at week-2 children enrolled will be seen by the study physician and base line safety tests will be performed and the visiting schedule determined including an agreement if the primary physician or study staff will see the patient at the clinical visits. Thereafter the study will commence week 0 which is noted as the study start date and immunizations will take place at weeks 0, 4, 12, and 36, the HAART treatment will be continued during the immunization and all the duration of the study. Additional visits are planned at weeks -4, 0, 4, 16, 40, 80. A study nurse will have weekly contact with the patient. Further clinical/study visits are planned for weeks -2, 4, 12, 20, 36, 40,48, 60, 72, and 96 in conjunction with regular visits. Blood letting for safety labs is performed at week -2, 0, 12, 16, 20, 36, 40, 48, 72, and 96 , where week 0,12, 20, 36, 48, 60 72 and 96 correspond to routine follow-up.

At total blood volume of 85 ml will be drawn for study purposes during the study time.

Termination of trial

Conclusion of the trial is scheduled at week 96. Patient meeting one of the previously defined endpoint as observed by the Trial Coordinating Committee, the Principal Investigator and the Study Monitor, are dropped out from the study.

The trial can be terminated at any time if the sponsor, the Medical Products Agency, or the Scientific Advisory Board, consider that there is a threat to the safety of the participants.

The investigators will draft and submit an amendment to this protocol so that patients randomised to HAART could be offered immunization, if one of these groups is judged superior, after they have initiated HAART and with at least 6 months of HIV RNA below 50.

Study organisation

Trial coordinating committee

Paolo Rossi MD,PhD (principal investigator, PI)

Stefania Bernardi,MD

Guido Castelli-Gattinara,MD

Britta Wahren MD, PhD

Paolo Palma MD, PhD student

Scientific Advisory Board

Gianfranco Bottazzo, MD, PhD

Viviana Moschese MD,PhD

Massimo Andreoni MD,Phd

DSMB- Safety Board

Eric Sandstrom MD,PhD

Clinical Trial Monitor

Chiara Mennini MD

Clinical trial staff

Stefania Bernardi, MD

Alessandra Martino, MD

Paolo Rossi MD, PhD

Giuseppe Pontrelli, MD

Hyppolite Tchidjou, MD

Paolo Palma, MD, PhD

Angela Aquilani, MD

Nadia Mora, MD

Giuseppe Pontrelli, MD

Veronica Santilli, MD

Laboratory staff

Maria Luisa Romiti Bsc, Phd

Simone Pensieroso Bsc, Phd

Claudia Capponi, Bsc

Carla Montesano, BsC, PhD

External collaborators

Gunnel Engstrom Bsc, PhD

Eric Sandstrom MD,PhD

Britta Wahren MD,PhD

Gunnel Biberfeld MD,Phd

Carlo Federico Perno, MD, PhD

Gabriella Scarlatti MD, PhD

Andreas Bråve PhD

Fabrizio Manca MD,PhD

Giuseppina Li Pira, PhD

Lindvi Gudmundsdotter PhD student

Stefania Dispinseri PhD student

<u>Safety</u>

All medical events should be recorded. They should be graded as to their seriousness,

severity and relationship to the immunization. For the purpose of this study all medical events are registered as adverse events. The investigator will assess and record each adverse event, whether or not considered drug related, in detail on the adverse event report form including the date and time of onset, description, severity, duration and outcome, aetiology, relationship of the adverse event to study drug, and action taken.

Patients with adverse events, including abnormal laboratory values, during the study or at the final visit must be treated and followed up according to established medical practice. The outcome of such treatment will be recorded in the CRF.

Immediate local (erythema, induration, pain, regional lymphadenopathy, restricted movement) and systemic (headache, nausea, fatigue, fever, gastrointestinal upset, vomiting, diarrhoea, myalgia, hypersensitivity reactions) reactions are directly observed during the first hour after the application and subsequently documented in a self-administered diary and reported in the CRF of each patient at scheduled visit time point.
Vital signs are recorded at each visit (heart rate, blood pressure, temperature, and weight)
Physical exam is performed prior to each injection and at the final visit.
Haematological and clinical chemistries are followed throughout the study (CBC with differential, platelets, ALT, creatinine, urinanalysis).

The severity of the event will for the purpose of this study be recorded as
mild if no action is required and the subject can continue ordinary activity,
moderate if some intervention is required, such as giving aspirin, but the subject can continue ordinary activity with some difficulty,
severe (the equivalent of grade 3 or 4) if the subject needs medical attention or is hospitalised or is unable to continue ordinary daily activity for a day or more.

All clinically significant abnormal values are to be identified by the investigator in the CRF.
The severity of the adverse event will be classified in accordance to the PACTG Toxicity grading scale.

The relationship of the adverse event to study drug will be assessed using the following definitions:
None
adverse events felt to be due to extraneous causes and neither follow a known pattern of response nor a reasonable temporal relationship to study product
Possible but unlikely

adverse events that are unlikely to be related to product but follow a reasonable temporal relationship, such that this cannot be completely excluded or events that could be associated with product but which are unrelated in time

Possible and likely

adverse events that may be due to extraneous causes but follow a known pattern of response and/or a reasonable temporal relationship to study product

Probable

adverse events that cannot be explained by extraneous causes; which follow a know pattern of response and/or a reasonable temporal relationship; which disappear or decrease on cessation of study product and reappear on rechallenge

The probability of a relationship of the adverse event to study drug will be assessed through the Jones' algorithm.

A serious adverse event is defined as any event that suggests a significant hazard, contraindication, side effect or precaution. With respect to human clinical experience, a serious adverse event includes any event that is fatal or life threatening, is permanently disabling, requires inpatient hospitalisation or is a congenital anomaly, cancer or overdose.

For **all grade 2 (that influence the immunizations), 3 and 4 or serious adverse events** should be recorded by the study personnel noticing it and a copy immediately forwarded to the Principal Investigator or his deputy.

The Principal Investigator shall evaluate the suspected causality and expectedness of the event. In case of any doubt the Scientific Advisory Board should be consulted or in any case of serious unexpected serious adverse event. The Scientific Advisory Board will in that case assist in breaking the code.

Any serious adverse even**t (including death) due to any cause or expectedness, which occurs during the course of this study, whether or not related to the investigational drug, must be reported by the Principal Investigator and the DSMB immediately to the sponsor who will, within 7 working days, inform the Scientific Advisory Board and the Trial Coordinating Committee.**

All unexpected grade 2 (that influence the immunizations), grade 3 and 4 adverse events that may be related to the immunizations shall be reported by the Principal Investigator and the to the sponsor who will inform Scientific Advisory Board, the Medical

products Agency within 15 days.

The Principal Investigator, will follow-up all grade 2 (that influence the immunizations), 3 and 4 or serious adverse events and supply that information to the sponsor and Scientific Advisory Board as soon as it becomes available.

In case the trial is temporarily or definitely halted a report should be made by the Principal Investigator to the Medical Products Agency and Scientific Advisory Board within 15 days.

In case serious unexpected serious adverse events or any other issues that may adversely affect the safety are reported from any source the Medical Products Agency and Scientific Advisory Board shall be informed within 15 days and ad interim analysis will be prontly performed.

A yearly report shall be submitted to the Medical Products Agency and Scientific Advisory Board containing a line listing of all grade 3 and 4 and serious adverse events with a commentary on the implication for the safety of the trial.

Data handling

Source data will be entered into the patients medical record according to clinical routine.

Study data will be collected under GCP conditions on single sheets of CRFs under code, kept in binders in a designated locked room and entered an apposite database in accord with the routines for patient records at Children’s Hospital “Bambino Gesù”. Laboratory data will be entered in this data bank as they become available. Extracts for analysis will only be done after personal identifiers have been removed.

Laboratory results will be kept under study code in the various laboratories and never combined with patient identity data.

Study data will be entered into a data base in Access under study code weekly. Any queries will be resolved and monthly a printout will be checked against the source data by an internal trial monitoring member.

At 3 monthly intervals the external monitor will review patient identity, the inclusion and exclusion criteria against the source data .

At 6 monthly intervals all the safety laboratory, CD4 and RNA data in the data base will be reviewed against source data by the external monitor and any discrepancies brought to the attention of the Trial Coordinating Committee.

The study physician or Principal Investigator can do this at any time for individual participants or the Trial Coordinating Committee for the entire trial by contacting the pharmacy. Officials from the Medical Products Agency or corresponding agencies may inspect the records and medical files for inconsistencies.
Original data will be kept for 15 years.

Ethical considerations The study will be conducted according to ICH GCP guidelines and the Declaration of Helsinki (version 2000).
The study involves a number of intramuscular immunizations and donations of blood. - The blood loss and pain of the investigations is judged to be reasonable compared to the potential gain in knowledge.

Data Ownership

The data generated in this study will be the property of the Investigators and will be held on their behalf by Children's Hospital "Bambino Gesù" and the Chair of Paediatrics of University of Rome "Tor Vergata", Swedish Institute for infectious Diseases Control has the right to obtain the full data set at any time during the trial and after closing the trial. If required at an earlier date for the purposes of regulatory submissions, a request should be made to the Trial Coordinating Committee.

Commercial interests Immunogens derive from Swedish Institutet for Infectious Disease Control and the Karolinska Institute. The plasmids products are produced by Vecura. The investigators are free to publish all the results from the studies after agreements with the owners.

Confidentiality and responsibilities, including indemnity

Full medical confidentiality will be preserved. The Principal Investigator is responsible for obtaining the appropriate Local Research Ethics Committee approval for the study protocol, the subject information sheet and the consent form. The Principal Investigator is responsible for informing the ethics committee of any serious adverse events as required, and submitting annual reports as required.
Vecura will be responsible for supply and labelling of the DNA plasmids.
Patients will be covered by "Bambino Gesù" Hospital insurance.

PROTOCOL AMENDMENTS

The Trial coordinating committee **(**TCC) prepares any amendments needed. Amendments to the protocol will be made only after consultation and agreement between the Principal

investigator (Paolo Rossi), and a sponsor SMI representative. The only exception is where the investigator considers that a subject's safety is compromised without immediate action. All amendments that have an impact on subject risk or the study objectives, or require revision of the informed consent document, must receive approval from the ethical committee prior to their implementation.

Publication It is intended that the results of this study will be published in an appropriate peer-reviewed journal, with the aim of submitting a paper for publication within 6 months of the study's completion. The collaborators will have 30 days to comment on any manuscript. No other publications, whether in writing or verbally, will be made before the definitive manuscript has been agreed and accepted for publication, without the prior approval of the TCC .

References

- Pensieroso P. Romiti ML, Palma P, et al. Switching from PI-based-HAART to PI-sparing Regimen is associated with improved specific HIV-immune responses in HIV-infected children. AIDS 2006,20: 1893-1896.

- Luzuriaga, ML Newell, F. Dabis et al Vaccines to prevent transmission of HIV-1 via breastmilk: scientific and logistical priorities K.. Lancet. 2006 Aug 5;368(9534):511-21.

- Lambert JS HIV vaccines in infants and children.Paediatr Drugs. 2005;7(5):267-76

- Jaspen LB.The maturing immune system: implications for development and testing HIV-1 vaccines for children and adolescents AIDS. 2006 Feb 28;20(4):483-94

- Legrand FA, Abadi J, Jordan KA, et al. Partial treatment interruption of protease inhibitors augments HIV-specif immune responses in vertically infected pediatric patients. AIDS 2005; 19:1575-1585.

- Palma P, Romiti M, Cancrini C, Successful simplification of protease inhibitor-based HAART with a triple nucleosides regimen in HIV-1 vertically infected children. AIDS 2007; Nov 21(18): 2465-2472 .

- Arnedo-Valero M, Garcia F, Gil C, Guila T, Fumero E, Castro P, Blanco JL, Miro JM, Pumarola T, Gatell JM. Clin Infect Dis. 2005 Sep 15;41(6):883-90.

- Brorsson B, Ifver J, Hays RD. The Swedish Health-Related Quality of Life Survey (SWED-QUAL). Qual Life Res. 1993 Feb;2(1):33-45.

- Calarota S, Bratt G, Nordlund S, Hinkula J, Leanderssson A-C, Sandström E, Wahren B.Cellular cytotoxicity induced by DNA vaccination in HIV-1-infected patients. Lancet 351:1320-1325, 1998

- Foli A, Maserati R, Barasolo G, Castelli F, Tomasoni L, Migliorino M, Maggiolo F, Pan A, Paolucci S, Scudeller L, Tinelli C, D'Aquila R, Lisziewicz J, Lori F. Strategies to decrease viral load rebound, and prevent loss of CD4 and onset of resistance during structured treatment interruptions. Antivir Ther. 2004 Feb;9(1):123-32.

- Garcia F, Plana M, Arnedo M, Ortiz GM, Miro JM, Lopalco L, Lori F, Pumarola T, Gallart T, Gatell JM. A cytostatic drug improves control of HIV-1 replication during structured treatment interruptions: a randomized study. AIDS. 2003 Jan 3;17(1):43-51.

- Hejdeman B, Bostrom AC, Matsuda R, Calarota S, Lenkei R, Fredriksson EL, Sandstrom E, Bratt G, Wahren B. DNA immunization with HIV early genes in HIV type 1-infected patients on highly active antiretroviral therapy. AIDS Res Hum Retroviruses. 2004 Aug;20(8):860-70.

- Lisziewicz J, Trocio J, Xu J, Whitman L, Ryder A, Bakare N, Lewis MG, Wagner W, Pistorio A, Arya S, Lori F. Control of viral rebound through therapeutic immunization with DermaVir. AIDS. 2005A Jan 3;19(1):35-43.

- Lisziewicz J, Trocio J, Whitman L, Varga G, Xu J, Bakare N, Erbacher P, Fox C, Woodward R, Markham P, Arya S, Behr JP, Lori F. DermaVir: a novel topical vaccine for HIV/AIDS. J Invest Dermatol. 2005B Jan;124(1):160-9.

- Lori F, Kelly LM, Foli A, Lisziewicz J. Safety of hydroxyurea in the treatment of HIV infection. Expert Opin Drug Saf. 2004 Jul;3(4):279-88. Review.
- Lova L, Groff A, Ravot E, Comolli G, Xu J, Whitman L, Lewis M, Foli A, Lisziewicz J, Lori F. Hydroxyurea exerts a cytostatic but not immunosuppressive effect on T lymphocytes. AIDS. 2005 Jan 8;19(2):137-144.
- Lu W, Arraes LC, Ferreira WT, Andrieu JM. Therapeutic dendritic-cell vaccine for chronic HIV-1 infection. Nat Med. 2004 Dec;10(12):1359-65. Epub 2004 Nov 28.
- Plana M, Garcia F, Oxenius A, Ortiz GM, Lopez A, Cruceta A, Mestre G, FumeroE, Fagard C, Sambeat MA, Segura F, Miro JM, Arnedo M, Lopalcos L, Pumarola T, Hirschel B, Phillips RE, Nixon DF, Gallart T, Gatell JM. Relevance of HIV-1-Specific CD4+ Helper T-Cell Responses During Structured Treatment Interruptions in Patients With CD4+ T-Cell Nadir Above 400/mm3. J Acquir Immune Defic Syndr. 2004 Jul 1;36(3):791-799.
- Sandstrom E, Wahren B. Therapeutic immunisation with recombinant gp160 in HIV-1 infection: a randomised double-blind placebo-controlled trial. Nordic VAC-04 Study Group. Lancet. 1999 May 22;353(9166):1735-42.
- Spetz AL, Sorensen AS, Walther-Jallow L, Wahren B, Andersson J, Holmgren L, Hinkula J. Induction of HIV-1-specific immunity after vaccination with apoptotic HIV-1/murine leukemia virus-infected cells. J Immunol. 2002 Nov 15;169(10):5771-9.
- Lindvi Gudmundsdotter & Anna Sjödin , Ann-Charlotte, Boström, Bo Hejdeman ,Rebecca Theve-Palm , Annette Alaeus , Knut Lidman , Britta Wahren Therapeutic immunization for HIV Springer Semin Immun (2006) 28:221–230
- Sekaly RP. The failed HIV Merck vaccine study: a step back or a launching point for future vaccine development? J.Exp Med. 2008 Jan 21;205(1):7-12.
- Flavia Ferrantelli ,Stefano Buttò, Aurelio Cafaro, Britta Wahren, Barbara Ensoli; Building collaborative networks for HIV/AIDS vaccine development: the AVIP experience.Springer Semin Immun (2006) 28:289.
- JJ. Donnelly, B. Wahren and M. A. Liu; DNA Vaccines: Progress and Challenges The Journal of Immunology, (2005), 175: 633-639.
- Li Pira G., Ivaldi F., Bottone L.High throughput functional microdissection of pathogen-specific T-cell immunity using antigen and lymphocyte arrays. J Immunol Methods. 2007,326,22-32.
- Li Pira et al. Miniaturization and automation for measuring T cell responses. Cytometry 2008, in press
- E Sandström, B Wahren, B Hejdeman, et al. Improved modes of delivering a safe and highly immunogenic multigene multiclade HIV-1 DNA plasmid vaccine boosted with HIV-1 MVA. In HIV pathogenesis and vaccines, Abstract Seattle 2007.

- Yerly S, Fagard C, Gunthard HF, Hirschel B, Perrin L; Swiss HIV Cohort Study. Antivir Ther. 2003 Oct;8(5):411-5

Pharmacy form 1

PEDVAC TRIAL VACCINE RANDOMIZATION REQUEST FORM
CLINICAL COPY

Date	Study number	Initials

**Name of the requesting personnel**	
**Signature**	

Randomized to ***Group A*** ***HAART*** ❒

Group B ***DNA-Vaccination+HAART*** ❒

Date ……………. Envelope number ……..

**Name of the randomizing personnel**	
**Signature**	

RETURN THIS FORM TO THE CLINIC

Pharmacy form 2
PEDVAC TRIAL VACCINE RANDOMIZATION,
PHARMACY COPY

Date	Study number	Initials

Date …………… Envelope number ……..

Name of the randomizing personnel	
**Signature**	

Randomized to ***Group A*** ***HAART*** ☐
Group B ***DNA-Vaccination+HAART*** ☐

Staple envelope content here marked with Study number, date and signature

ARCHIVE IN THE PHARMACY BINDER

Pharmacy form 3

PEDVAC TRIAL VACCINE REQUISITION FORM

Date	Study number	Initials

Immunization number **1** ☐ **2** ☐ **3** ☐ **4** ☐

**Name of the requesting personnel**	
**Signature**	

Vaccine dispensed ***Date…………….. Time……………***

**Name of the dispensing personnel**	
**Signature**	

Vaccine Received in clinic ***Date…………….. Time……………***

**Name of the recipient in clinic**	
**Signature**	
